# Genetic Architecture of Aluminum Tolerance in Rice (*Oryza sativa*) Determined through Genome-Wide Association Analysis and QTL Mapping

**DOI:** 10.1371/journal.pgen.1002221

**Published:** 2011-08-04

**Authors:** Adam N. Famoso, Keyan Zhao, Randy T. Clark, Chih-Wei Tung, Mark H. Wright, Carlos Bustamante, Leon V. Kochian, Susan R. McCouch

**Affiliations:** 1 Department of Plant Breeding and Genetics, Cornell University, Ithaca, New York, United States of America; 2 Department of Biological Statistics and Computational Biology, Cornell University, Ithaca, New York, United States of America; 3 Robert W. Holley Center for Agriculture and Health, Agricultural Research Service, US Department of Agriculture, Cornell University, Ithaca, New York, United States of America; The University of North Carolina at Chapel Hill, United States of America

## Abstract

Aluminum (Al) toxicity is a primary limitation to crop productivity on acid soils, and rice has been demonstrated to be significantly more Al tolerant than other cereal crops. However, the mechanisms of rice Al tolerance are largely unknown, and no genes underlying natural variation have been reported. We screened 383 diverse rice accessions, conducted a genome-wide association (GWA) study, and conducted QTL mapping in two bi-parental populations using three estimates of Al tolerance based on root growth. Subpopulation structure explained 57% of the phenotypic variation, and the mean Al tolerance in *Japonica* was twice that of *Indica*. Forty-eight regions associated with Al tolerance were identified by GWA analysis, most of which were subpopulation-specific. Four of these regions co-localized with *a priori* candidate genes, and two highly significant regions co-localized with previously identified QTLs. Three regions corresponding to induced Al-sensitive rice mutants (*ART1*, *STAR2*, *Nrat1*) were identified through bi-parental QTL mapping or GWA to be involved in natural variation for Al tolerance. Haplotype analysis around the *Nrat1* gene identified susceptible and tolerant haplotypes explaining 40% of the Al tolerance variation within the *aus* subpopulation, and sequence analysis of *Nrat1* identified a trio of non-synonymous mutations predictive of Al sensitivity in our diversity panel. GWA analysis discovered more phenotype–genotype associations and provided higher resolution, but QTL mapping identified critical rare and/or subpopulation-specific alleles not detected by GWA analysis. Mapping using *Indica*/*Japonica* populations identified QTLs associated with transgressive variation where alleles from a susceptible *aus* or *indica* parent enhanced Al tolerance in a tolerant *Japonica* background. This work supports the hypothesis that selectively introgressing alleles across subpopulations is an efficient approach for trait enhancement in plant breeding programs and demonstrates the fundamental importance of subpopulation in interpreting and manipulating the genetics of complex traits in rice.

## Author Summary

While rice (*Oryza sativa*) is significantly more Al tolerant than other cereals, no genes underlying Al tolerance in rice have been reported. Using genome-wide association (GWA) and bi-parental QTL mapping, we investigated the genetic architecture of Al tolerance in rice. *Japonica* varieties were twice as Al tolerant as *indica* and *aus* varieties. Overall, 57% of the phenotypic variation was correlated with subpopulation, consistent with observations that different genes and genomic regions were associated with Al tolerance in different subpopulations. Four regions identified by GWA co-localized with *a priori* candidate genes, and two highly significant regions co-localized with previously identified quantitative trait loci (QTL). Haplotype and sequence analysis around the candidate gene, *Nrat1*, identified a susceptible haplotype explaining 40% of the Al tolerance variation within the *aus* subpopulation and three non-synonymous mutations within *Nrat1* that were predictive of Al sensitivity. Using *Indica *×* Japonica* mapping populations, we identified QTLs associated with transgressive variation where alleles from a susceptible *indica* or *aus* parent enhanced Al tolerance in a tolerant *japonica* background. This work demonstrates the importance of subpopulation in interpreting and manipulating complex traits in rice and provides a roadmap for breeders aiming to capture genetic value from phenotypically inferior lines.

## Introduction

Aluminum (Al) toxicity is the major constraint to crop productivity on acid soils, which comprise over 50% of the world's arable land [1]. Under highly acidic soil conditions (pH<5.0), Al is solubilized into the soil solution as Al^3+^, which is highly phytotoxic, causing a rapid inhibition of root growth that leads to a reduced and stunted root system, thus having a direct effect on the ability of a plant to acquire both water and nutrients.

Cereal crops (*Poaceae*) have been a primary focus of Al tolerance research [2]. This research has demonstrated that levels of Al tolerance vary widely both within and between species [3]–[8]. Of the major cereal species that have been extensively studied (rice, maize, wheat, barley and sorghum), rice demonstrates superior Al tolerance under both field and hydroponic conditions [3], [8]. Although rice is 6–10 times more Al tolerant than other cereals, very little is known about the genes underlying this tolerance. Based on its high level of Al tolerance and numerous genetic and genomic resources, rice provides a good model for studying the genetics and physiology of Al tolerance.

In wheat, sorghum, and barley, Al tolerance is inherited as a simple trait, controlled by one or a few genes [9]–[11]. However, in maize, rice, and *Arabidopsis*, tolerance is quantitatively inherited [12], [13]. Al tolerance genes have been cloned in wheat and sorghum. The wheat resistance gene, *ALMT1*, encodes an Al-activated malate transporter [14]. The sorghum resistance gene, *SbMATE*, encodes a member of the multidrug and toxic compound- extrusion (MATE) family and is an Al-activated, root citrate efflux transporter [15]–[17].

Four mutant genes that lead to Al sensitivity in rice have recently been cloned, *STAR1* (*Sensitive to Al rhizotoxicity1*), *STAR2* (*Sensitive to Al rhizotoxicity2*), *ART1* (*Aluminum rhizotoxicity 1*), and *Nrat1* (*Nramp aluminum transporter 1*) [18]–[20]. The products of *STAR1* and *STAR2* are expressed mainly in the roots and are components of a bacterial-type ATP binding cassette (ABC) transporter. Both are transcriptionally activated by exposure to Al and loss of function of either gene results in hypersensitivity to Al. *STAR1* and *STAR2* are similar to two Al sensitive mutants in *Arabidopsis*, *als1* and *als3*, also encoding ABC transporters [21], [22]. *ART1* is a novel C2H2-type zinc finger transcription factor that interacts with the promoter region of *STAR1*. *ART1* is reported to regulate at least 30 down-stream genes, some of which are involved in Al detoxification and serve as strong candidate genes controlling rice Al tolerance [19]. *Nrat1* is one of the genes that is regulated by *ART1* and was recently demonstrated to be an Al transporter that is localized to the root cell plasma membrane [18], [20]. It is hypothesized that *Nrat1* confers Al tolerance by transporting Al into the cell and reducing the concentration of Al in the cell wall [20]. None of the four cloned rice genes described above have been demonstrated to be involved in natural genetic variation of Al tolerance in rice and only one (*Nrat1*) maps to a previously reported Al tolerance QTL [23], suggesting that these genes may be involved in basal Al tolerance [19], [20], [24]. A more thorough analysis is necessary to determine whether there might be natural variation associated with these loci that would help trace their evolutionary origins and clarify their contribution to the high levels of Al tolerance observed in rice.

Seven QTL studies on Al tolerance have been reported in rice using 6 different inter- and intra-specific mapping populations [13], [25]–[29]. Together, these studies report a total of 33 QTLs, located on all 12 chromosomes, with three intervals (on chromosomes 1, 3, and 9) being detected in multiple studies. In all of the QTL studies, Al tolerance was estimated based on relative root growth (RRG), and specifically on inhibition of the growth (elongation) of the longest root (elongation of the longest root in Al treatment/root growth of controls). Rice has a very fine and fibrous root system without dominant seminal roots. We recently showed that there is a weak correlation between rice Al tolerance based on RRG of the longest root and RRG of the total root system (R^2^ = 0.17) [8]. This raises the question whether mapping Al tolerance QTL using total root and longest root RRG indices independently might identify novel loci, helping to integrate QTL studies with studies based on induced mutations.

Historically, *O. sativa* has been classified into two varietal groups, *Indica* and *Japonica*, based on morphological characteristics, ecological adaptation, crossing ability and geographic origin [30]. These two varietal groups are believed to represent independent domestications from a pre-differentiated ancestral gene pool (*O. rufipogon*), followed by significant gene flow among and between subpopulations [17], [31]–[39]. These two varietal groups (names are italicized with an upper case first letter, i.e., *Indica* and *Japonica*) have been further divided into five major subpopulations (subpopulation names are italicized using all lower-case letters) (*indica*, *aus*, *tropical japonica*, *temperate japonica*, and *aromatic [group V]*) based on DNA markers (SSR, SNPs, indels) [40]–[42]. Genotypes that share <80% ancestry across subpopulations or varietal groups are classified as admixed varieties [42], while smaller groups adapted to specific ecosystems may be recognized as upland, deep water, or floating varieties [43], [44]. Upland varieties, which are generally grown at high altitudes on dry (non-irrigated) soils, are those most commonly exposed to acidic, Al-toxic soil conditions. These varieties are almost invariably of *tropical japonica* origin, suggesting *a priori* that the *tropical japonica* subpopulation would be a likely source of superior alleles for Al tolerance in rice.

Diverse panels of *O. sativa* are reported to have similar, or slightly elevated levels of linkage disequilibrium (LD) compared to species such as Arabidopsis, maize and human. The average extent of LD in rice has been estimated at between 50–500 kb [45]–[49], depending on the germplasm evaluated, compared to 10–250 kb in Arabidopsis and human [50]–[57], 100–500 kb in commercial elite maize inbreds and 1–2 kb in diverse maize landraces [58], [59]. The inbreeding nature of *O. sativa*, coupled with its demographic history, are major determinants of genome-wide patterns of LD. Strong selective pressure over the course of rice domestication has also lead to deep population substructure (F_st_ = 0.23 to 0.57) [40], [42], which sets it apart from Arabidopsis, in which population structure is gradual across geographic distances [60], [61]. Population substructure can lead to false-positives in association mapping studies, and must be taken into account [61]–[63]. The mixed-model has been demonstrated to work well in both maize and Arabidopsis [61], [63], and it has also shown its ability to greatly reduce the false positive rates in rice when used within a single subpopulation [64], though it may introduce false negatives when used on a diversity panel representing all domesticated subpopulations [65].

A diversity panel consisting of 413 *O. sativa* accessions, representing the genetic diversity of the primary gene pool of domesticated rice [66], was recently genotyped with 44,000 SNPs (∼10 SNPs/kb) [65], [67], [68] as the basis for GWA studies. The slow decay of LD, while facilitating GWA analysis, limits the resolution of association mapping in rice. The first targeted association mapping study in rice [45] demonstrated that LD decay in the *aus* subpopulation was approximately 90 kb (∼5 genes) in a region on chromosome 5 containing the *xa5* resistance gene. LD is expected to decay more quickly in *O. rufipogon* (<50 kb, or 1–3 genes) [48], providing higher resolution for LD mapping, and more slowly in the *japonica* subpopulations [47]–[49]. Nonetheless, when compared to the resolution of a typical QTL study (250 lines) (∼10–20 cM resolution, where 1 cM = ∼250 kb), association mapping is expected to provide between 10–200 times higher resolution for a population of similar size as long as sufficient marker density is obtained to exploit the historical recombination. Thus, an association mapping study that uses markers densities similar to a QTL study will not have the increased resolution and will increase the risk of type-2 error. For both GWA and QTL analysis in rice, fine-mapping and/or mutant analysis is generally required to identify the gene(s) underlying a QTL of interest. However, the fine-mapping phase can generally be focused on a smaller target region following GWA analysis.

In this study, the genetic architecture of rice Al tolerance was investigated via bi-parental QTL analysis in two mapping populations using relative root growth of the longest root, the primary root system, and the total root system quantified with the digital root phenotyping methods described previously for rice Al tolerance [8]. Subsequently, genome wide association (GWA) analysis was undertaken using 36,901 high quality SNPs that had been genotyped on the rice diversity panel [65]. Regions identified by GWA were compared with regions identified as QTLs in bi-parental mapping populations for both this and previous studies, as well as with Al sensitive mutants and/or candidate genes. Phenotypic outliers identified in the diversity panel were further investigated to identify regions of subpopulation-admixture that accounted for extreme Al tolerance phenotypes.

## Results

### Al Tolerance in Rice

**Figure 1:**
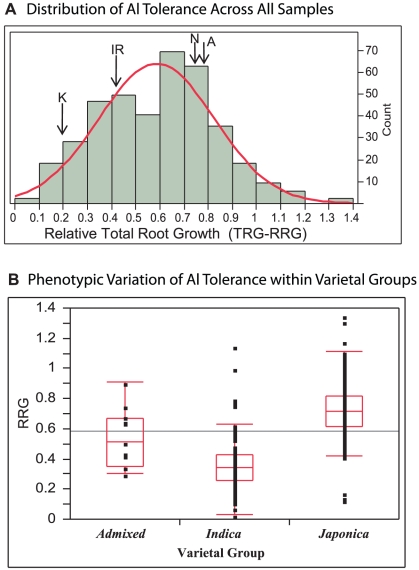
Distribution of Al Tolerance in Rice Diversity Panel. A) Distribution of Al tolerance across 383 diverse accessions of *O. sativa* at 160 µM Al^3+^. Aluminum tolerance (TRG-RRG) was normally distributed around a mean of 0.59 +/−0.24(SD) and ranged from 0.03–1.35. The Al tolerance of the QTL mapping parents are indicated: K = Kasalath, IR = IR64, N = Nipponbare, A = Azucena. B) Variation of Al tolerance (RRG) within genetic varietal groups (>80% ancestry). Admixed accessions share <80% ancestry with either group. The *Japonica* varietal group (*temperate* and *tropical japonica* and *aromatic* subpopulations) is significantly more tolerant than the *Indica* varietal group (*indica* and *aus* subpopulations) (p<0.0001). Five *Indica* accessions were identified to be highly Al tolerant outliers and six *Japonica* outlier accessions were identified, three as highly Al susceptible and three as highly tolerant.

Three hundred eighty three diverse *O. sativa* accessions from the rice diversity panel [42], [67](Table S1) were evaluated for Al tolerance using an Al^3+^ activity of 160 µM in a hydroponic nutrient solution. This Al^3+^ activity had been previously determined to be optimal for evaluating a wide range of Al tolerance in diverse rice germplasm [8]. In the diversity panel, Al tolerance, measured as the relative root growth of the total root system (TRG-RRG), was normally distributed around a mean of 0.59 +/−0.24(SD) and ranged from 0.03–1.35 (Figure 1A). Some varieties were inhibited by as much as 97%, while 16 varieties (representing three subpopulations) showed enhanced root growth in the presence of 160 µM Al^3+^ (Table S1).

When accessions were grouped based on varietal group (>80% ancestry) the *Japonica* varietal group (consisting of the *temperate japonica*, *tropical japonica* and *aromatic* subpopulations) was significantly more Al tolerant than the *Indica* varietal group (*indica* and *aus* subpopulations) (p<0.0001) (Figure 1B). The *Japonica* varieties had a mean Al tolerance value of RRG = 0.72, an interquartile range of 0.61–0.82, and ranged from 0.13–1.35. The *Indica* varieties had a mean Al tolerance value of RRG = 0.36, an interquartile range of 0.27–0.43, and ranged from 0.03–1.15 (Figure 1B). Eleven accessions were classified as “admixed” between varietal groups, and these had a mean Al tolerance equal to the mean of all 372 accessions (TRG-RRG = 0.59) with >80% ancestry to either varietal group. A one-way ANOVA demonstrated that subpopulation explained 57% of the phenotypic variation observed for Al tolerance (TRG-RRG) among the 274 accessions that carried a subpopulation classification. Despite the differences in mean TRG-RRG between subpopulations, considerable variation was also detected within each subpopulation (Figure S1).

### QTL Analysis

Two immortalized QTL mapping populations were analyzed for Al tolerance. One consisted of 134 recombinant inbred lines (RIL) derived from the cross IR64/Azucena [69], and the other was comprised of 78 backcross inbred lines (BIL) derived from the cross Nipponbare/Kasalath//Nipponbare [70]. These populations were used to evaluate Al tolerance using three different indices of relative root growth (RRG), (1) longest root growth (LRG-RRG), (2) primary root growth (PGR-RRG) and (3) total root growth (TRG-RRG) (see Materials and Methods for details). The phenotypic distribution was approximately normal for each population, no matter which root screening index was used (illustrated for TRG-RRG in Figure S2A and S2B). The QTL mapping populations allowed us to determine which of the three root evaluation methods would be most useful for evaluating the diversity panel as a whole.

**Figure 2:**
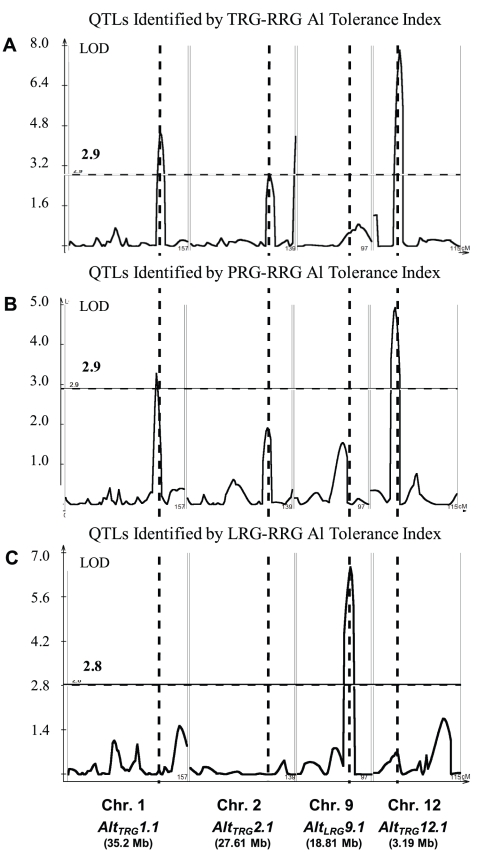
QTLs Identified in IR64 × Azucena RIL Mapping Population. A–C) Composite interval mapping output for QTL detected in the RIL mapping population using three Al tolerance RRG indices. The Y-axis is the LOD score and the horizontal line is the significant LOD threshold based on 1000 permutations. QTL name and approximate physical position are along bottom of figure and co-localization of QTLs identified with different Al tolerance indices are indicated with dashed vertical lines. A) Total root growth (TRG-RRG); B) Primary root growth (PRG-RRG); C) Longest root growth (LRG-RRG).

The method of phenotyping, specifically, the RRG index used to estimate Al tolerance, directly impacted the significance of QTLs detected by composite interval mapping (Figure 2A–2C and Figure S3A–S3C). In the RIL population, three Al tolerance (*Alt*) QTL were detected using total root growth (the TRG-RRG index), *Alt*_TRG_ *1.1* on chromosome 1, *Alt*_TRG_ *2.1* on chromosome 2, and *Alt*_TRG_ *12.1* on chromosome 12 (Figure 2A–2C Table 1). The Azucena allele conferred increased tolerance at the loci on chromosomes 1 and 12 and reduced tolerance at the locus on chromosome 2. QTLs were detected in the same positions on chromosomes 1 and 12 using RRG based on primary root growth (the PRG-RRG index), although with lower LOD scores (Figure 2A–2C; Table 1). Using longest root growth (the LRG-RRG index), a single QTL was detected on chromosome 9, *Alt*_LRG_ *9.1*, and this QTL was not detected when the other root indices were used. The major QTL on chromosome 12 (*Alt*_TRG_ *12.1*), which explained >19% of the variation in Al tolerance based on TRG-RRG, is located between 2.69–5.10 Mb and encompasses the Al sensitive rice mutant *art1*, which is located at 3.59 Mb [19].

**Table 1:** Summary of significant QTLs (1000 permutations) identified by composite interval mapping in the RIL and BIL populations.

Trait Index	Population	Chr.	QTL	Peak Marker	Peak Mb Position	Flanking Markers	LOD1 L (Mb)	LOD1 R (Mb)	LOD	Additive effect	R^2^
TRG-RRG	RIL	1	*Alt_TRG_ 1.1*	RM265	35.2	RM319/RM315	34.32	36.10	4.56	2.58 (Azu)	0.095
PRG-RRG	RIL	1	*Alt_PRG_ 1.1*	RM265	35.2	RM319/RM315	34.36	35.93	3.29	3.84 (Azu)	0.081
TRG-RRG	BIL	1	*Alt_TRG_ 1.2*	RM6333	38	RM5448/RM8231	37.70	38.68	3.44	−10.58 (Nip)	0.117
TRG-RRG	RIL	2	*Alt_TRG_ 2.1*	RM221	27.61	RM526/RM318	26.79	29.17	2.9	−2.08 (IR64)	0.059
PRG-RRG	BIL	6	*Alt_PRG_ 6.1*	L688	5.81	R1954/G200	2.82	6.67	3.95	12.78 (Kas)	0.143
LRG-RRG	RIL	9	*Alt_LRG_ 9.1*	RM242	18.81	RM257/RM160	18.15	19.40	6.57	4.42 (Azu)	0.165
TRG-RRG	RIL	12	*Alt_TRG_ 12.1*	RM247	3.19	RM453/RM512	2.88	3.89	7.85	3.76 (Azu)	0.193
PRG-RRG	RIL	12	*Alt_PRG_ 12.1*	RM247	3.19	RM453/RM512	2.75	4.54	4.94	4.75 (Azu)	0.126
TRG-RRG	BIL	12	*Alt_TRG_ 12.2*	R2708	23.36	R1709/G2140	22.33	25.00	3.49	12.3 (Kas)	0.128

Al tolerance (RRG) QTLs were identified using three root growth parameters, total root growth (TRG), primary root growth (PRG), and longest root growth (LRG). The parent contributing the tolerance allele is indicated in parentheses under additive effect.

In the BIL population, two QTL were detected using the TRG index, *Alt*_TRG_ *1.2* on chromosome 1, which co-localized with the *Alt*_TRG_ *1.1* QTL identified in the RIL population, and *Alt*_TRG_ *12.2* on chromosome 12, which did not overlap with the *Alt*_TRG_ *12.1* identified in the RIL population (Figure 2A–2C, Figure S3A–S3C, Table 1). The Nipponbare allele conferred tolerance at the chromosome 1 locus and the Kasalath allele conferred tolerance at the *Alt*_TRG_ *12.2* locus. No QTLs were detected on chromosome 2 in the BIL population. Using the PRG-RRG index, one QTL was detected on chromosome 6, where the Kasalath allele conferred resistance. No QTLs were detected using the LRG-RRG index in the BIL population.

The Al tolerance index used for evaluating the phenotype directly affected both the identity and the significance of the QTLs detected. Al tolerance index-specific QTLs were detected in both populations and no QTL locus was detected across all three indices. Based on number of QTL detected, significance of QTL, and variance explained by the QTL, total root growth (TRG) proved to be the single most powerful Al tolerance index. However, rice QTLs detected using different evaluation methods are likely to confer Al tolerance by different mechanisms, such as tolerance of primary, secondary, lateral, or all roots, and thus they are complementary and together provide a robust evaluation of the genetic architecture of Al tolerance than any single index alone.

### Identification of Al Tolerance Loci through GWA Mapping

**Figure 3:**
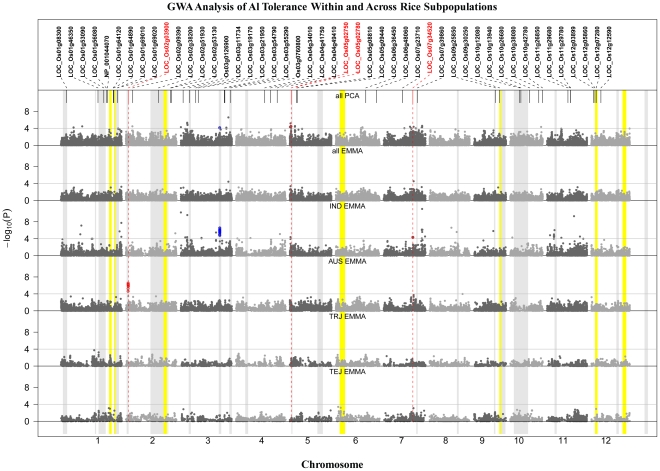
GWA Analysis of Al Tolerance within and across Rice Subpopulations. GWA analysis across and within subpopulations (IND* = indica*; AUS* = aus*; TRJ = *tropical japonica*; TEJ = *temperate japonica*). *A priori* candidate genes are listed across the top, with those identified within 200 kb of significant SNPs colored red. Color bands indicate the 23 bi-parental QTL positions from previous reports (grey) or from this study (yellow). SNP color indicates co-localization with QTLs (blue) or candidate genes (red).

To identify Al tolerance loci based on genome-wide association (GWA) mapping, we used an existing genotypic dataset consisting of 36,901 SNPs [65], and the total root growth (TRG-RRG) Al tolerance phenotype generated on 373 *O. sativa* accessions over the course of this study. GWA mapping was conducted, using SNPs with a MAF>0.05, across all 373 genotypes as well as independently within the *indica*, *aus*, *temperate japonica*, and *tropical japonica* subpopulations (Figure 3). The Efficient Mixed-Model Association (EMMA) [71] model was used in each analysis (both within and across subpopulations) to correct for confounding effects due to subpopulation structure and relatedness between individuals. As the subpopulation structure was highly correlated with Al tolerance, it was observed that analyzing all samples (373) together with the EMMA model resulted in an overcorrection (causing type 2 error) and a corresponding reduction in SNP significance (Figure S4). To address this problem, a PCA approach was also employed when analyzing all (373) samples together. However, the PCA approach resulted in a slight under-correction for population structure (Figure S4), demonstrating that results from each GWA method has limitations when used across all germplasm in this highly structured diversity panel.

A total of ∼48 distinct Al tolerance genomic regions were identified by GWA mapping (Figure 3). Twenty-one regions were detected (p<0.0001) across all (373) accessions using the PCA model (Figure 3), while only two SNPs were above the significance threshold when all (373) accessions were analyzed together using the EMMA model (Figure 3), both of which were also detected by PCA. The threshold of p<1.0E-04 was determined based on the upper-limit false discovery rate (FDR), determined from the candidate genes in the same approach as in Li et al. [72] (Table S2). Thirty-two regions were significantly associated with Al tolerance in the *indica* subpopulation (Figure 3), including five regions that were also detected across all (373) samples using the PCA model. In the *aus* subpopulation, a single, highly significant, region was detected on chromosome 2 that was unique to this subpopulation and contained the *Nrat1* candidate gene LOC_Os02g03900 (Figure 3). No significant SNPs (MAF>0.05) were detected in the *temperate japonica* or *tropical japonica* subpopulations. The GWA mapping results indicate that the majority of significant loci are subpopulation-specific and that phenotypic variation for Al tolerance within given subpopulations is largely controlled by alleles that are unique to that subpopulation.

SNPs identified by GWA were also compared to a set of 46 *a priori* candidate genes as well as to positions of QTL regions identified through bi-parental mapping (this study and previous reports) (Table 1 and Figure 3). Two regions of highly significant SNP clusters, one within the *aus* (8 SNPs; p = 2.8E-07) subpopulation on chr. 2 and one within the *indica* (32 SNPs; p = 2.9E-07) subpopulation on chr. 3, co-localized to previously reported QTLs in populations in which an *aus* and *indica* parent served as the susceptible parents, respectively [17], [23]. The list of 46 *a-priori* Al tolerance candidate genes (Table 2) was compiled based on published information on Al sensitive mutants from rice and Arabidopsis [20]–[22], [24], cloned Al tolerance genes from wheat and sorghum [14], [15], expression profiles from Al treated maize and rice roots [19], [73], and an association study on specific candidate Al tolerance genes of maize [74]. Significant SNPs (p<1.0E-04) within a 200 kb window of the *a priori* candidate genes were enriched 2.4 times compared to other SNPs (p>0.0001) outside of the *a priori* and QTL regions. The 200 kb window was selected to fall within the estimated window of LD decay in rice (∼50–500 kb [45]–[49] and the upper-limit false discovery rate for the *a priori* genes was 42%. In addition, four of the 46 gene candidates (∼9%) were located within a 200 kb window enriched for GWA SNPs in this study (Figure 3 and Table 2). One of the candidate genes (*Nrat1*) on chr. 2, co-localized with both GWA SNPs and a previously reported QTL (Figure 3). The relationship between the four candidates that co-localized with GWA SNPs are discussed in order of their positions on the rice genome below.

**Table 2:** List of 46 *a priori* Al tolerance candidate genes.

	LOC ID	Reference	Chr.	Mb Pos.	(Homolog) Description	GWA detection	p-value
1	LOC_Os01g178300	[19]	1	4.07	OSCDT3		
2	LOC_Os01g46350	[19]	1	26.37	proteins of unknown function		
3	LOC_Os01g53090	[19]	1	30.51	pathogen-related protein, putative		
4	LOC_Os01g56080	[19]	1	32.28	expressed protein		
5	LOC_Os01g64120	[19]	1	37.24	2Fe-2S iron-sulfur cluster binding		
6	LOC_Os01g64890	[19]	1	37.66	CorA-like magnesium transporter		
7	LOC_Os01g69010	[15]	1	40.09	(SbMATE) MATE efflux protein		
8	LOC_Os01g69020	[19]	1	40.10	retrotransposon protein, putative		
9	NP_001044070	[19]	1	33.05	SAM-dependen methyltransferase		
10	LOC_Os02g03900	[19], [20]	2	1.66	(Nrat1) metal transporter Nramp6	AUS	4.99E-07
11	LOC_Os02g09390	[19]	2	4.82	cytochrome P450, putative		
12	LOC_Os02g38200	[74]	2	23.10	dehydrogenase, putative, expressed		
13	LOC_Os02g51930	[19]	2	31.80	cytokinin-O-glucosyltransferase 2		
14	LOC_Os02g53130	[19]	2	32.51	nitrate reductase, putative, expressed		
15	LOC_Os03g11734	[74]	3	6.13	MATE efflux protein		
16	LOC_Os03g19170	[19]	3	10.75	GCRP7 - Glycine and cysteine rich		
17	LOC_Os03g21950	[74]	3	12.54	fumarate hydratase		
18	LOC_Os03g54790	[19], [21], [24]	3	31.14	(ALS1) ABC transporter, ATP-binding protein		
19	LOC_Os03g55290	[19]	3	31.46	GASR3 - Gibberellin-regulated		
20	Os03g0760800	[19]	3	35.66	GA-regulated protein family		
21	Os03g0126900	[19]	3	1.75	hypothetical protein		
22	LOC_Os04g34010	[74]	4	20.42	(ALMT1) aluminum-activated malate transporter		
23	LOC_Os04g41750	[19]	4	24.56	expressed protein		
24	LOC_Os04g49410	[19]	4	29.30	expansin precursor		
25	LOC_Os05g02750	[22], [24]	5	0.99	(ALS3 and STAR2) ABC transporter	All-PCA	3.5E-05
26	LOC_Os05g02780	[74]	5	1.00	glycine-rich protein A3, putative	All-PCA	3.5E-05
27	LOC_Os05g08810	[74]	5	4.85	phosphatidylinositol 3-kinase		
28	LOC_Os05g09440	[74]	5	5.29	malic enzyme		
29	LOC_Os06g36450	[74]	6	21.40	ferroportin1 protein		
30	LOC_Os06g48060	[19], [24]	6	29.07	(STAR1) ABC transporter, ATP-binding		
31	LOC_Os07g23710	[74]	7	13.38	cytochrome P450, putative		
32	LOC_Os07g34520	[74]	7	20.69	isocitrate lyase	IND	4.49E-05
33	LOC_Os07g39860	[19]	7	23.90	expressed protein		
34	LOC_Os09g25850	[19]	9	15.49	WAX2, oxidoreductase;		
35	LOC_Os09g30250	[19]	9	18.41	OsSub58 - Putative Subtilisin		
36	LOC_Os10g12080	[74]	10	6.73	cytochrome P450, putative		
37	LOC_Os10g13940	[19]	10	7.59	MATE efflux protein		
38	LOC_Os10g26680	[74]	10	13.86	pectinesterase, putative, expressed		
39	LOC_Os10g38080	[19]	10	20.32	OsSub61 - Putative Subtilisin homologue		
40	LOC_Os10g42780	[19]	10	23.00	lrgB-like family protein, expressed		
41	LOC_Os11g26850	[74]	11	14.96	erythronate-4-phosphate dehydrogenase		
42	LOC_Os11g29680	[19]	11	16.74	expressed protein		
43	LOC_Os11g29780	[19]	11	16.82	plant-specific domain TIGR01627		
44	LOC_Os12g03899	[74]	12	1.61	major facilitator superfamily		
45	LOC_Os12g05860	[74]	12	2.69	Cupin domain containing protein		
46	LOC_Os12g12590	[19]	12	6.93	NADP-dependent oxidoreductase		

Genes identified within 200 kb of SNPs detected by GWA analysis (p<1.0E-0.4) are indicated. GWA detection refers to the germplasm set in which the region was identified (IND = *indica*; All-PCA = all samples).

A cluster of eight highly significant SNPs (p-values = 2.3×10^−5^–2.8×10^−7^) on chromosome 2 between 1.536 Mb–1.675 Mb was associated with Al tolerance within the *aus* subpopulation (Figure 3 and Table 2). Previously, a QTL had been reported in the same location (0.536–1.9 Mb) where the susceptible parent was of *aus* origin [26]. The LD decay in the *aus* subpopulation at this region was calculated to be 150 kb and a strong candidate gene was identified within the target region. The gene (LOC_Os02g03900 located at 1.66 Mb) encodes a Nramp6 metal transporter and was demonstrated to have altered expression patterns in Al-treated roots of the Al sensitive *art1* rice mutant [19]. This Nramp6 metal transporter was recently reported as *Nrat1*, a plasma membrane-located transporter for Al with enhanced sensitivity to Al in the knockout mutant [20]. As was the case with the *ART1* gene itself, the *Nrat1* metal transporter has not been associated with natural variation for Al tolerance prior to this study.

On chromosome 5, a significant region was detected across all samples (373 genotypes) by PCA, co-localizing with the *STAR2* gene (LOC_Os05g02750) (Figure 3 and Table 2). The LD decay across this region was estimated at >500 kb, and encompassed two significant regions detected across all samples (PCA), one of which was also detected within the *indica* subpopulation. *STAR2* is the rice ortholog of the *Arabidopsis* Al sensitive mutant *als3* [21]. It encodes the transmembrane domain of a bacterial-type ATP binding cassette (ABC) transporter and the *star2* mutant is Al sensitive [24]. *STAR2* was also found to be part of a gene network showing altered expression in response to Al in the *art1* mutant compared to the *ART1* wild type [19]. This study provides the first evidence that there may be natural variation for Al tolerance in rice at the *STAR2* locus; however it is important to recognize that the PCA approach may under-correct for the effect of subpopulation in this study, thus it will be necessary to confirm the effect of the *STAR2* alleles identified in this diversity panel.

A significant GWAS region identified in the *indica* subpopulation on chromosome 7 co-localized with LOC_Os07g34520, a rice ortholog of a maize isocitrate lyase *a priori* candidate gene associated with Al tolerance in maize [73], [74]. The LD decay across this region within the *indica* subpopulation was 250 kb.

Three highly significant regions detected within *indica* were further investigated to identify whether any clear Al tolerance candidate genes were located within these SNP clusters. The first region was a cluster of 32 significant SNPs (p = 3.0E-7) between 28.782–27.863 Mb on chr. 3 that co-localized with a previously reported QTL (Nguyen et al., 2002). Two clear candidates were identified among the 13 genes in this cluster; a nucleobase-ascorbate transporter (LOC_Os03g48810) and a chloride channel protein (LOC_Os03g48940). The second region was a 10 SNP cluster (p = 9.3E-12) between 26.986–27.479 Mb on chr. 7. Of the 80 genes in this region, 34 of which were retrotransposons, there were three strong candidate genes; a glycosyl transferase protein (LOC_Os07g45260), a cytochrome P450 protein (LOC_Os07g45290) and a zing finger RING type protein (LOC_Os07g45350). This region on chr. 7 was also identified in the introgression analysis as a localized introgressed region from *Japonica* into the highly tolerant *Indica* outliers (discussed below). The third region was an 8 SNP cluster between 4.892–5.164 Mb on chr. 11. Among the 48 genes in this region, there were two major classes of candidate genes observed, including 12 F-box proteins and a zinc finger CCHC protein.

### Haplotype Analysis of *Nrat1* Gene Region on Chromosome 2

**Figure 4:**
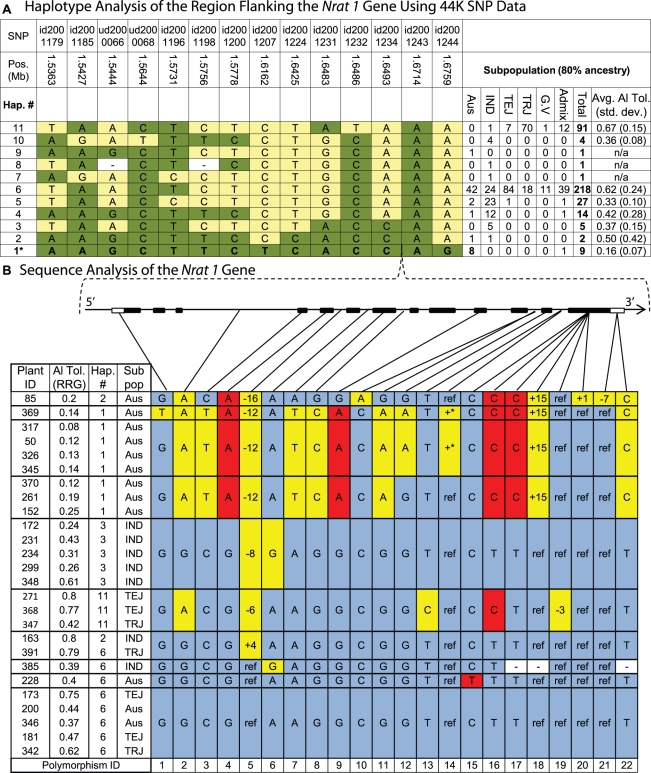
Haplotype analysis of the *Nrat1* gene region. A) Haplotypes observed in 373 accessions using the 44,000 SNP data. Haplotype 1 was unique to *aus* ancestry and associated with Al susceptibility within the *aus* subpopulation, explaining 40% of the Al tolerance variation within *aus.* Haplotypes 1, 2, and 3 share the same 4-SNP haplotype (id2001231-id2001243) flanking the *Nrat1* gene (1.66 Mb). SNP positions are based on MSU6 annotation and subpopulations are abbreviated as follows: IND = *indica*, TEJ = *temperate japonica*, TRJ = *tropical japonica*, G.V. = *groupV*/*aromatic*, Admix = admixed lines without 80% ancestry to any one subpopulation. B) Haplotypes at the *Nrat1* gene (1.66 Mb) in the (9) *aus* and (6) *indica* accessions sharing the 4-SNP haplotype flanking the *Nrat1* gene. Polymorphisms are identified with numbers along bottom of figure. A STOP codon occurs in exon 13 between polymorphism 17 and 18. Gray shaded cells represent the reference allele and plant ID# 173 is the reference genotype ‘Nipponbare’. Yellow shaded cells represent polymorphisms in introns or synonymous polymorphisms in exons. Red shaded cells represent polymorphisms that result in amino acid substitutions (Indel or non-synonymous), unshaded cells marked with “−” indicate missing data, and +* indicates an intron insertion >500 bp.

We chose to further investigate the variation in and around the *Nrat1* gene on chromosome 2 because multiple independent lines of evidence supported the existence of a gene(s) in this region responsible for a significant portion of the variation for Al tolerance in rice. Evidence included a strong GWA peak in the *aus* subpopulation, a previously reported QTL [26], and the localization of the *Nrat1* Al transporter gene. Using the 44 K SNP data, LD in this region was calculated to be ∼150 kb in the *aus* subpopulation and 11 distinct haplotypes were observed in the entire diversity panel across a 139 kb region around the *Nrat1* gene (1.536 Mb–1.675 Mb on chr. 2) (Figure 4A). Haplotype 1 (Hap. 1), which was unique to the *aus* subpopulation, was found in 8 Al sensitive *aus* accessions and one Al sensitive *aus/indica* admixed line. These 9 genotypes were among the least Al tolerant (7^th^ percentile, mean RRG = 0.16) of the 373 accessions screened (Table S1). Haplotype 1 explained 40% of the phenotypic variation for Al tolerance within the *aus* subpopulation (Figure S5). In addition, four *aus* accessions that were highly or moderately Al tolerant were found to contain a *tropical japonica* introgression across this region (described in the section on Introgression analysis below).

Haplotype 2 (Hap. 2) was found in one *aus* and one *indica* accession, and was most similar to Hap. 1, differing at only 2/14 SNPs (Figure 4A). The two lines containing haplotype 2 had very different levels of Al tolerance; the *aus* variety, Kasalath (ID 85), was highly susceptible, with a RRG = 0.2, while the *indica* variety, Taducan (ID 163), was tolerant, with a RRG = 0.8, suggesting that this extensive 14-SNP haplotype across the 139 kb region was not predictive of Al tolerance. However, when the haplotype was built using only the four SNPs immediately flanking the *Nrat1* gene, a group of 16 accessions sharing the same haplotype at these four SNPs was clearly identified. These 16 accessions, included the 10 susceptible *aus* accessions (including one *aus/indica* admixed line) carrying haplotype 1 and haplotype 2 and six *indica* accessions (of varying Al tolerance) carrying haplotype 2 and haplotype 3 (Figure 4A).

To determine if the four-SNP haplotype flanking the *Nrat1* gene could be further resolved, we focused more deeply on the *Nrat1* gene itself. We sequenced all 13 exons (including introns) of *Nrat1* (1874 bp) in 26 susceptible and tolerant varieties representing the *aus*, *indica*, *tropical japonica* and *temperate japonica* subpopulations (Figure 4B). The accessions carried haplotypes 1, 2, 3, 6 and 11, as described in Figure 4A; where haplotype 1 was *aus-*specific and corresponded to the most sensitive group of accessions in the diversity panel; haplotype 2 was found in phenotypically divergent *aus* and *indica* accessions as described above; haplotype 3 was found in moderately tolerant *indica* varieties; haplotype 6, which appeared to be the ancestral haplotype, was the most common haplotype in all subpopulations and was associated with moderately high levels of tolerance; and haplotype 11, which was found in a majority of *tropical japonica* varieties, all of which were Al tolerant. Based on the 22 SNPs and/or indels identified across the 1,874 bp of *Nrat1* sequence, highly resolved, gene haplotypes were constructed (Figure 4B). The gene haplotypes corresponded fairly well to the extended haplotype groups that had been constructed using the data from the 44 K SNP chip, except in the case of haplotype 2, where varieties differed at 10/22 (45%) of the SNPs across the *Nrat1* gene. This fully resolved haplotype at the *Nrat1* gene resulted in the susceptible Kasalath clustering with the other highly susceptible *aus* varieties and the tolerant Taducan clustering with other highly tolerant varieties (Figure 4).

Three non-synonymous SNPs (polymorphisms 4, 16, 17) were shared among the 9 highly susceptible *aus* accessions. When the Eukaryotic Linear Motif resource (http://elm.eu.org) was used to identify functional sites in the *Nrat1* gene, polymorphism 16 was identified as a functional site where a C→T SNP caused an amino acid change from valine→alanine (amino acid 500). This protein site was predicted to be involved in PKA-type AGC kinase phosphorylation, with the functional site spanning amino acids 497–503. Thus, polymorphism 16 was identified as a strong functional polymorphism candidate underlying natural variation in *Nrat1*. The fact that polymorphism 16 was also observed in two Al tolerant *temperate japonica* and one moderately tolerant *tropical japonica* accession (haplotype 11) suggested that SNP 16 alone was not predictive of Al tolerance. However, a combination of polymorphisms 4, 16, and 17 was entirely predictive of Al susceptibility.

This study demonstrates the power of whole genome association analysis to integrate divergent pieces of evidence from independent bi-parental and mutant studies, enabling us to associate gene-based diversity with germplasm resources and natural variation that is of immediate use to plant breeders.

### Introgression Analysis

There is a clear difference in the degree of Al tolerance found in the *Japonica* varietal group and the *Indica* varietal group, with the 10^th^ percentile of Al tolerance of *Japonica* (0.53) being nearly equal to the 90^th^ percentile of *Indica* (0.55) (Figure 1B). However, there are clear outliers within each varietal group. Five *Indica* accessions are highly Al tolerant (ID 30, 66, 142, 163, 337), ranging from 2.1–3.2 times the mean *Indica* Al tolerance, and three *Japonica* accessions (ID 12, 52, 112) are highly susceptible, each approximately 0.19 of the mean *Japonica* Al tolerance (Figure 1B and Table S1).

To determine if these outliers were the result of introgressions across varietal groups, we calculated the allele ancestry of 5,467 SNPs distributed throughout the genome and identified specific genomic regions where historical *Indica×Japonica* admixture was detected only in the respective *Indica* or *Japonica* outlier lines. To do this, *Japonica* introgressions identified in highly Al tolerant *Indica* lines were used to query all other *Indica* accessions and only those *Japonica* introgressions that were uniquely present in the highly Al tolerant outlier *Indica* lines were considered as candidate regions underlying the outlier phenotype. When the five *Indica* outliers were used for this analysis, a few, well-defined regions comprising 2.4–4.9% of the genome corresponded to regions of *Japonica* introgression (Table 3). In the case of the three highly Al susceptible *Japonica* varieties, the genetic background was highly heterogeneous and the small number of lines precluded doing any admixture analysis. Therefore, the admixture analysis was conducted only on the five highly tolerant *Indica* outliers.

**Table 3:** Summary of *Japonica* introgressions in the *Indica* outliers.

Chr.	Introgres- sion I.D.	Line #	Introgression (MSU6 Mb pos.)	Size (Mb)	GWA Signal	Previous QTL
1	1.1	30, 163	41.69–42.06	0.37	IND	none
2	2.1	66, 142^a^	21.93–23.10	1.17	none	Nguyen V, 2001
7	7.1	30^b^, 66^c^, 142^d^, 163^e^	27.05–27.62	0.57	IND	none
8	8.1^f^	30, 142, 163	0.032–0.42	0.39	none	none
8	8.2	30, 163^g^	7.61–7.82	0.21	none	Nguyen V, 2002
11	11.1	30, 66, 163^h^	19.06–20.05	0.99	IND	none

*Indica* outliers ranged from 94.6–97.6% *Indica* ancestry throughout the genome. Six regions were identified where the outliers shared unique introgressions from *Japonica* that were observed only in Al tolerant *Indica* outliers and were not present in any other *Indica*. Five of the six introgressed segments encompass regions identified in GWA analysis or bi-parental QTL analysis. Three introgressed regions encompass SNPs identified within the *indica* (IND) subpopulation, across all subpopulations (All), or both. Two of the introgressions encompass previously reported QTLs.

a Line 142: introgression 2.1 is 21.93–23.80 Mb and TRG-RRG = 1.15.

b Line 30: introgression 7.1 is 27.05–29.65 Mb and TRG-RRG = 0.76.

c Line 66: introgression 7.1 is 27.05–27.62 Mb and TRG-RRG = 1.00.

d Line 142: introgression 7.1 is 27.05–29.65 Mb and TRG-RRG = 1.15.

e Line 163: introgression 7.1 is 25.98–29.65 and TRG-RRG = 0.80.

f Introgression 8.1 is a novel locus that does not co-localize with GWA or QTL loci.

g Line 163: introgression 8.2 is 7.61–10.14 Mb and TRG-RRG = 0.80.

h Line 163: introgression 11.1 is 18.43–20.05 Mb and TRG-RRG = 0.80.

In the five outlier *Indica* accessions, 6 *Japonica* introgressions (median size = 780 kb) were identified that were specific only to these 5 lines. Three of these introgressions were present in two genotypes, two of the introgressions were present in three genotypes, and one introgression was present in four of the outliers (Table 3). Three introgressions encompass SNPs identified by GWA analysis and two co-localized with bi-parental QTL. The introgression that was present in four of the *indica* outlier genotypes was located on chromosome 7 between 27.05–28.62 Mb and contained 94 annotated genes. This introgression included a cluster of GWA SNPs that were highly significant within the *indica* subpopulation (p = 2.6×10^−5^, MAF = 0.10) and was one of the top 100 most significant SNPs identified when the diversity panel as a whole was analyzed.

## Discussion

### Utilization of GWA and Bi-Parental QTL Mapping

In this study, we utilized bi-parental QTL mapping and GWA analysis to examine the genetic architecture of Al tolerance in rice and to identify Al tolerance loci. Phenotyping of the diversity panel provided valuable information about the range and distribution of Al tolerance in *O. sativa* and offered new insights into the evolution of the trait. The mean Al tolerance in *Japonica* was twice that of *Indica* (p<0.0001), and 57% of the phenotypic variation was explained by subpopulation. The relative degree of Al tolerance in the five subpopulations (*temperate japonica>tropical japonica>aromatic>indica = aus*) was consistent with the level of genetic relatedness among them [42], [44] and suggests that *temperate* and *tropical japonica* germplasm contain alleles that would be useful sources of genetic variation for enhancing levels of Al tolerance within *indica* and *aus*. This is supported by the identification of highly tolerant *indica* varieties from the rice diversity panel that contain introgressions from *Japonica* in regions characterized by GWA peaks. The highly tolerant *Indica* outliers demonstrate the feasibility of using a targeted approach to increase Al tolerance in *Indica* varieties by introgressing genes from *Japonica*.

While less obvious, our QTL analysis demonstrated the ability to increase Al tolerance in *Japonica* using targeted introgressions from *Indica*. This was demonstrated within both QTL populations by the identification of two loci in which alleles from the highly susceptible Kasalath parent conferred enhanced levels of Al tolerance in the Nipponbare genome (*temperate japonica*) and one locus where the moderately susceptible IR64 parent conferred enhanced tolerance in crosses with Azucena (*tropical japonica*) (Table 1). To date, only a few *indica* and *aus* accessions have been used in QTL mapping populations and the identification of a large number of GWA loci in *indica*, coupled with the fact that *indica* is significantly more diverse than all other *O. sativa* subpopulations [40], [42] suggests that there are likely to be many novel alleles that could be mined from the *indica* subpopulation. Further evidence of the value of this approach in the context of plant breeding comes from the transgressive variation observed in both QTL populations, where some RILs and BILs exceeded the Al tolerance observed in the tolerant *tropical* and *temperate japonica* parents, Azucena and Nipponbare, respectively, due to alleles derived from the susceptible *indica* (IR64) or *aus* (Kasalath) parents, respectively.

The significant differences in Al tolerance among varietal groups and subpopulations, and evidence that different genes and/or alleles contribute to Al tolerance within the major varietal groups, is consistent with *Indica* and *Japonica* domestication from pre-differentiated, wild *O. rufipogon* gene pools that differed in Al tolerance. Future experiments will test this hypothesis by comparing levels of Al tolerance found in wild populations of *O. rufipogon*. The inherently higher levels of Al tolerance found in the *Japonica* varietal group may help explain why *tropical japonica* varieties are so often found in the acid soils of upland environments.

Compared to QTL mapping, GWA significantly increases the range of natural variation that can be surveyed in a single experiment and the number of significant regions that are likely to be identified. Furthermore, GWA provides higher resolution than QTL mapping, facilitating fine-mapping and gene discovery. This was illustrated by the two highly significant regions detected by GWA that overlapped with previously reported QTLs. GWA detected a highly significant cluster of 32 SNPs (p = 2.9E-07) on chr. 3 within the *indica* subpopulation, defining the candidate region to 81 kb window containing 13 genes, while the previously reported QTL interval was 1,720 kb [17], containing 260 genes. Similarly, the *Nrat1* locus identified within the *aus* subpopulation on chromosome 2 initially narrowed the target region to 139 kb containing 27 genes by GWA, while the previously reported [26] QTL interval was 1,360 kb and contained 234 genes.

Surprising, the *Nrat1* region was not significant in the BIL population, in which the resistant parent (Nipponbare) contained a resistant haplotype at *Nrat1* and the susceptible parent (Kasalath) contained the susceptible haplotype at *Nrat1*. The fact that a significant signal was not detected in the BIL population can likely be explained by one or more of the following: 1) the bias inherent in the small population size (78 BILs), 2) the backcross population structure in which only 11 individuals (14% of BILs) contained the Kasalath allele at the *Nrat1* locus and/or 3) the effects of genetic background on the *Nrat1* QTL region. The *Nrat1* QTL region was detected in one previous QTL study by Ma et al. [23] where a BIL population consisting of 183 lines was used, with Kasalath as the susceptible *aus* parent and Koshihikari as the tolerant *temperate japonica* parent [23]. In that study, the *Nrat1* QTL region was of minor significance (LOD = 2.81; R^2^ = 7%), and it is noteworthy that the two other (more significant) QTLs detected in that study were the two QTLs detected in our BIL population using only 78 lines. The fact that the *Nrat1* QTL region was not detected in our BIL mapping population and was of low significance in the Ma et al. QTL study suggests that the effect of the Kasalath allele is likely to be influenced by genetic background effects (GXG). In an *aus* genetic background, the *Nrat1* susceptible haplotype explains 40% of the phenotypic variation, and the diversity panel contains enough *aus* varieties for this to be statistically significant using GWA; however, in the BIL population where Nipponbare served as the recurrent parent, the *aus* alleles exist in a largely *temperate japonica* background. Given the extent of GXG observed in inter-sub-population crosses, and the small size of our BIL population, this appears to be the most likely explanation as to why the *Nrat* locus was not detected in our QTL experiment.

Although GWA significantly increased the power and resolution of QTL detection, nearly all the significant loci detected were subpopulation-specific. This is entirely consistent with the strong subpopulation structure in rice and the high correlation of Al tolerance with subpopulation, justifying our GWA analysis on each subpopulation independently. So the question might be asked as to why it is also necessary to conduct GWA in the diversity panel as a whole? The answer to this question lies in the complex biology and demographic or breeding history of *O. sativa.* In this study GWA was conducted both within and across subpopulations, and it demonstrated that GWA on the diversity panel as a whole leveraged power to detect alleles that were segregating across multiple subpopulations, even if they were rare within any one subpopulation group, while when used on independent subpopulations, it was useful in detecting alleles that segregated only within one or two subpopulations but tended to be fixed in others. This is what would be expected from what we know about the evolutionary history of rice with its examples of shared domestication alleles [35], [75] coupled with myriad subpopulation-specific alleles [41], [48], [76]–[78] that provide each subpopulation with its specific identity and spectrum of ecological adaptations.

There are cases in which QTLs discovered by bi-parental mapping are not detected by GWA analysis. One reason for this is that QTL mapping can readily detect alleles that are rare in a diversity panel, are subpopulation-specific, or where the phase of the allelic association differs across subpopulations, while GWA analysis has limited power to do so. This is important in the case of rice, because of the degree of differentiation between the subpopulations and the significant evolutionary differences between the *Indica* and *Japonica* varietal groups, as discussed above. Thus, while variation that is strongly correlated with subpopulation structure is undetectable by GWA analysis, these loci can be easily detected by QTL analysis if crosses between sub-populations are used. This is illustrated by the identification of the Al tolerance QTL, (*Alt*_TRG_ *12.1*) encompassing the *ART1* locus on chromosome 12. This large-effect QTL (LOD = 7.85, R^2^ = 0.193) was clearly detected in the RIL population but was not detected by GWA analysis. The QTL mapping populations utilized in this study were of limited population size and thus largely underpowered [79]. As a result it is likely that some QTL effects were overestimated and that other small effect QTL were not detected. Although we cannot be certain of the exact amount of variance explained by a particular QTL, it is reasonable to conclude that the major QTL detected (*Alt*_TRG_ *12.1*) is, in fact, the most significant QTL in the population.

GWA mapping also provides a valuable link between functional genomics and natural variation, and in the case of rice, highlights the subpopulation-specific distribution of specific alleles and phenotypes. We implicate the involvement of the *STAR2* (chr. 6)/*ALS3* (*Arabidopsis* Al sensitive mutant) gene, previously identified as induced mutations in rice and *Arabidopsis*, respectively [22], [23], and document the detection of highly resolved, novel Al tolerance loci in the *indica* and *aus* subpopulations. This is a critical bridge for germplasm managers and plant breeders who look for alleles of interest in germplasm collections rather than as sequences in GenBank.

### Analysis of *Nrat1* Gene

Our strongest example of the value of linking functional genomics and natural variation is illustrated by the GWA region on chromosome 2, where we demonstrate that the *aus-*specific susceptible haplotype in this region is functionally related to an *Nramp* gene. This gene was previously identified to have altered expression in the *art1* (transcription factor) Al sensitive mutant [19] and was recently reported as *Nrat1* (for *Nramp aluminum transporter*), an Al transporter localized to the plasma membrane of root cells, which when knocked out, enhances Al susceptibility. This is consistent with this transporter serving to mediate Al uptake by moving it directly into root cells, presumably into the vacuole, and away from the root cell wall [20]. Our haplotype analysis of the GWA region on chromosome 2 and sequence analysis of the *Nrat1* gene identified putative sensitive and tolerant haplotypes that implicate the *Nrat1* gene, and further identified two putative functional polymorphisms specific to the Al sensitive *aus* accessions. These data provides valuable information for identifying *Nrat1* alleles that can be used to test the hypothesis put forth by Xia et al. [20], namely that Al tolerance is conferred by reducing Al concentrations in the cell wall. It will be interesting to see if the sensitive alleles of this gene encode an Nramp transporter that is less effective at mediating Al uptake. Furthermore, the observation that three of the four most Al tolerant *aus* accessions contain *tropical japonica* introgressions across this gene region strongly suggests that Al tolerance of *aus* genotypes can be increased by the targeted introgression of *tropical japonica* DNA at the *Nrat 1* region.

### Phenotyping Methods Affect QTL Detection

One of the objectives of this study was to determine if the Al tolerance index employed (longest root growth [LRG], primary root growth [PRG], or total root growth [TRG]) affected the detection and/or significance of Al tolerance QTL. In a recent publication from our research team, it was demonstrated that significantly different Al tolerance scores were obtained with the different indices [8]. In all previous QTL studies, Al tolerance was determined based on relative root growth (RRG) of the longest root. This study demonstrated that the Al tolerance index has a direct effect on the detection and significance of QTLs. Total root growth (TRG) was the single most powerful Al tolerance index, based on number of QTL detected, significance of QTL and variance explained by the QTL. However, it is relevant to point out that LRG-RRG identified a large-effect QTL (*Alt*_LRG_9.1) in the RIL population that was not detected using any other index, and PRG-RRG identified a unique QTL on chromosome 6 where the susceptible Kasalath variety carried the resistance allele. These observations suggest that different root evaluation methods are likely to identify Al tolerance QTLs that confer tolerance mediated by different types of roots, or possibly by different patterns of gene expression detectable only when specific phenotypic evaluation protocols are used.

The strongest example of the importance of utilizing the TRG-RRG index is demonstrated by the identification of the *Alt*_TRG_ *12.1* QTL in the RIL mapping population. The *ART1* gene, a C2H2-type zinc finger-type transcription factor that causes Al hypersensitivity when mutated, is located close to the center of the *Alt12.1* QTL peak. When this gene was first identified, it was suggested that it was not involved in natural variation of Al tolerance in rice, as no QTL had ever been identified in the region [19]. Based on our results, it is likely that this QTL was not previously identified because relative root growth was measured only based on LRG, rather than on TRG-RRG. Further fine-mapping of this locus, along with sequence and expression analysis, is underway to determine whether the *ART1* locus underlies this QTL and to understand the mechanism by which it contributes to natural variation for Al tolerance.

Previous studies in other cereals have reported that the correlation of Al tolerance between hydroponics and field conditions is >70% [80] and studies on rice Al tolerance mutants have demonstrated that tolerance/susceptibility observed in hydroponics screens is also observed under soil conditions [24]. To accurately assess the value of the loci detected in this study as targets of selection in rice breeding programs, we are currently developing experiments to determine the effect of the key loci detected in this work under Al-toxic field conditions. Furthermore, four sets of reciprocal NILs (8 NILs total) for the four QTLs detected in the RIL population are being developed to determine the effect of each QTL under both hydroponic and field conditions. Finally, field experiments will be conducted to determine which hydroponic root measurement phenotype (TRG, PRG, or LRG) is the best for predicting a genotypes Al tolerance under field conditions.

### Implications for Rice Breeding

This study provides the most comprehensive analysis of the genetic architecture of Al tolerance in rice to date. It demonstrates the power of whole genome association analysis to identify phenotype-genotype relationships and to integrate disparate pieces of evidence from QTL studies, mutant analysis, and candidate gene evaluation into a coherent set of hypotheses about the genes and genomic regions underlying quantitative variation. By tracing the origin of Al tolerance alleles within and between rice subpopulations, we provide new insights into the evolution and combinatorial potential of different alleles that will be invaluable in breeding new varieties for acid soil environments. This work demonstrates how genetic and phenotypic diversity is partitioned by subpopulation in *O. sativa* and provides support for the hypothesis that the most efficient approach to enhancing many quantitative traits in rice is to selectively introgress genes/alleles from one subpopulation into another. Our study also lays the foundation for understanding the genetic basis of Al tolerance mechanisms that enable rice to withstand significantly higher levels of Al than do other cereals. It not only facilitates more efficient selection of tolerant genotypes of rice, but it points the way toward using this knowledge to enhance levels of Al tolerance in other plant species.

## Materials and Methods

### Plant Growth Conditions and Germplasm

Plants were grown hydroponically in a growth chamber as described by Famoso et al. [8]. Al tolerance was determined based on relative root growth (RRG) after three days in Al (160 µM Al^3+^) or control solution. The hydroponic solution used in this study was chemically designed and optimized for rice Al tolerance screening; for a detailed comparison of the phenotypic procedures employed in this work compared to previously published rice Al tolerance work see Famoso et al. (2010). To obtain uniform seedlings, 80 seeds were germinated and the 30 most uniform seedlings were visually selected and transferred to a control hydroponic solution for a 24 hour adjustment period. After the 24 hour adjustment period, root length was measured with a ruler and the 20 most uniform seedlings were selected and distributed to fresh control solution (0 uM Al^3+^) or Al treatment solution (160 uM Al^3+^). Plants were grown in their respective treatments for ∼72 hours and the total root system growth was quantified using an imaging and root quantification system as described by Famoso et al. (2010). The mean total root growth was calculated for Al treated and control plants and RRG was calculated as mean growth (Al)/mean growth (control). The 373 genotypes screened for Al tolerance and used in the association analysis are part of a set of 400 *O. sativa* genotypes that have been genotyped with 44,000 SNPs as described by Zhao et al. [65].

### QTL Analysis and Heritability

The QTL populations consisted of a population of 134 recombinant inbred lines (RILs) derived from a cross between Azucena (tolerant *tropical japonica*) and IR64 (susceptible *indica*) [67], [70] and a population of 78 backcross introgression lines (BILs) derived from a cross between Nipponbare (tolerant *temperate japonica*) and Kasalath (susceptible *aus*) and backcrossed to Nipponbare. The Al^3+^ activity at which Al tolerance was screened was determined by identifying the Al^3+^ activity that provided the greatest difference in tolerance between the parents. The tolerant parent of the RIL population, Azucena, and the tolerant parent of the BIL population, Nipponbare, are similar in Al tolerance, whereas the susceptible parent of the RIL population, IR64, is significantly more tolerant than the susceptible parent of the BIL population, Kasalath (Figure 1A). To ensure that a normal distribution was obtained in each population, a different Al^3+^ concentration was used for each mapping population. The RIL population was screened at 250 µM Al^3+^ because the Azucena parent is very Al tolerant and the IR64 parent is only moderately susceptible. The BIL population was screened at 120 µM Al^3+^ because the Kasalath parent is extremely Al sensitive, though the Nipponbare parent is very Al tolerant. Figure 1 displays the Al tolerance of each mapping parent in reference to the 373 genetically diverse rice accessions screened at 160 µM Al^3+^. The genetic component of the phenotypic variance was calculated as VarG = VarG+Var(GxE)+error. QTL analysis was conducted using composite interval mapping (CIM) function in QTL Cartographer [81]. The significance threshold was determined by 1000 permutations.

### Genome-Wide Association Analysis

Genome-Wide Association Analysis was performed using three approaches in all samples (373) with phenotypes. The first approach was the naïve approach, which is simply the linear regression of phenotype on the genotype for each SNP marker. The second approach was principle component analysis (PCA), where we obtained the four main PCs (principle components) that reflect the global main subpopulations in the sample to correct population structure estimated from software EIGENSOFT. [82]. The first four PCs are included as cofactors in the regression model to correct population structure: 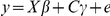.

Here β and γ are coefficient vectors for SNP effects and subpopulation PCs respectively. 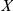 and 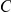 are the corresponding SNP vector and first 4 PC vectors, and 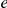 is the random error term. The third approach was the linear mixed model proposed by [62], [63], implemented in the R package EMMA [71], which models the different levels of population structure and relatedness. The model can be written in a matrix form as: *y = Xβ*+*Cγ*+*Zμ*+*e* where β and γ are the same as above, both of which are fixed effects, and 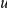 is the random effect accounting for structures and relatedness, 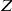 is corresponding design matrices, and 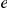 is the random error term. Assume *μ*∼*N(0,σ^2^_g_K)* and *e*∼*N(0,σ^2^_e_I)*, and K is the IBS matrix, as in [62]. We also conducted GWA using both the naïve approach and the mixed model approach in each of the four main subpopulations (IND, AUS, TEJ, TRJ). For the mixed model, the model was changed to *y = Xβ*+*Zu+e*, since there was no main subpopulation division within each subpopulation sample. Linkage disequilibrium decay and haploblocks were calculated at specific chromosome/gene regions using Haploview software [83].

### Admixture Analysis

Population structure was analyzed employing Expectation-Maximization techniques on an HMM model of per-marker ancestry along a chromosome with a weak linkage model between adjacent markers on the same chromosome induced by the HMM's state dependence on the previous marker's subpopulation assignment (M. Wright, Cornell University, personal communication). The 5,467 SNPs used for admixture analysis were a subset of the 36,901 high quality SNPs on the 44 K chip, and were selected based on their information content and ability to distinguish genetic groups, rather than individuals. The two main criteria used to select the subset of SNPs were a) good genomic distribution and minimal LD among those used in the analysis, and b) MAF>0.05 in at least one subpopulation. The state of the HMM at each marker corresponds to the subpopulation of origin for the marker (and by extension, the region containing the marker and its adjacent markers). The number of *a priori* distinct subpopulations was K = 5, consistent with that reported previously by Garris et al. 2005 and Ali et al., 2011 [40], [66]. A set of 50 standard non-admixed “control” lines, 10 representing each of the Garris et al. subpopulations, that were genotyped on the 44 K rice SNP array were used to develop and evaluate the method. All 50 lines were correctly assigned to each of the subpopulations and concordant with previous results using STRUCTURE [84], with little or no admixture or introgressions detected. The EM/HMM method was favored over the corresponding “linkage model” of recent versions of STRUCTURE because the EM/HMM model explicitly modeled inbreeding and estimated the inbreeding coefficient for each line independently, permitting lines in various stages of purification or inbreeding to homozygosity to be analyzed. The lines phenotyped in this study that were also genotyped on the 44 K SNP array were then analyzed, combined with these 50 control lines and the local ancestry along chromosomes were assigned by maximizing the state path of the HMM while simultaneously estimating subpopulation specific allele frequencies using the forward-backward algorithm. Using this method, introgressions from a foreign subpopulation into a line with a vast majority of the genetic background originating from a single subpopulation were detected.

## Supporting Information

Figure S1 Distribution of Al tolerance (TRG-RRG) by subpopulation (>80% ancestry). Subpopulation explains 57% of phenotypic variation, however significant variation exist within each subpopulation. IND = *indica*, TEJ = *temperate japonica*, TRJ = *tropical japonica*, G.V. = *groupV*/*aromatic*, Admix = admixed lines without 80% ancestry to any one subpopulation. Phenotypic outliers were detected within the *indica* (five tolerant, one susceptible), *temperate japonica* (one tolerant, one susceptible), and *tropical japonica* (two tolerant) subpopulations.
(EPS)

Figure S2 Distribution of Al tolerance in RIL and BIL mapping populations. A) Al tolerance (TRG-RRG at 250 µM Al^3+^) observed in 134 RILs derived from Azucena (tolerant *tropical japonica*) and IR64 (susceptible *indica*). The RIL population had a mean TRG-RRG of 39%, with a range of 21–67%. Under control conditions, the genetic component of phenotypic variation was 0.46, while in the Al^3+^ treatment, the genetic component of phenotypic variation was 0.35. Transgressive segregation was observed in 20% of the RILs, with 10% of the population demonstrating greater Al tolerance than Azucena (the tolerant parent) and 10% demonstrating greater susceptibility than IR64 (the susceptible parent). Three Al tolerant outliers were observed in the RIL population. B) Distribution of TRG-RRG Al tolerance at 120 µM Al^3+^ observed in 78 BILs derived from Nipponbare (tolerant *temperate japonica*) and Kasalath (susceptible *aus*). The BIL population had a mean TRG-RRG value of 73%, with a range of 45–120%. In control conditions, the genetic component of phenotypic variation was 0.45 while in the Al^3+^ treatment, the genetic component of phenotypic variation was 0.55. Transgressive segregation was only observed for increased Al tolerance, as no BIL was more susceptible than the Kasalath parent. One Al tolerant outlier was observed in the BIL population and the Kasalath parent was an Al susceptible outlier.
(EPS)

Figure S3 Composite interval mapping in the BIL mapping population using three Al tolerance RRG indices. The Y-axis is the LOD score and the horizontal line is the significant LOD threshold based on 1000 permutations. A) Total root growth; B) Primary root growth; C) Longest root growth.
(EPS)

Figure S4 Quantile–Quantile plot comparing p-values for the mixed model, PCA, and naïve models. Grey dashed line represents the null distribution. Colored solid lines of the observed ordered −log10(p-value) on the Y-axis vs expected log10(p-value) on the X-axis from bottom to top correspond to different methods: Mixed model, PCA and Naïve. The Naïve model does not correct for subpopulation structure or relatedness, resulting in highly inflated −log10 p-values. The PCA model accounts for major subpopulation structure, but not the more subtle correlation among accessions within subpopulation (measured as Identical By State matrix), resulting in a slight inflation of observed −log10 p-values, while the Mixed Model resulted in a slight overcorrection of subpopulation structure and a reduction in the observed −log10 p-values.
(EPS)

Figure S5 Oneway ANOVA for Al tolerance within the *aus* subpopulation (55 accessions). The presence/absence of the susceptible haplotype flanking the *Nrat1* gene region in the *aus* subpopulation explained 40% of the phenotypic variation for Al tolerance in the *aus* subpopulation.
(EPS)

Table S1 Aluminum tolerance and subpopulation identity of 383 genotypes from the rice diversity panel. Ten genotypes denoted with asterisk (*), did not have existing SNP genotype data at the time of GWA analysis and were not included in the GWA analysis. Subpopulation ancestry was based off 80% identity: AUS = *aus*; IND = *indica*; TRJ = *tropical japonica*; TEJ = *temperate japonica*; Group V is also known as *aromatic*. Any line with less than 80% subpopulation identity was considered an admixture (ADMIX). The two major varietal groups are *Indica* and *Japonica*; the *Indica* varietal group is comprised of the *aus* and *indica* subpopulations and the *Japonica* varietal group is comprised of the *temperate japonica*, *tropical japonica*, and *group V* subpopulations.
(DOC)

Table S2 Evaluation criteria for selecting candidate SNPs based on P-values from EMMA within and across subpopulations and *a priori* knowledge of candidate genes. SNPs within a 200 kb window around 46 *a priori* candidate genes were considered *a priori* SNPs. *Other SNPs* were those that fell outside of the 200 kb window surrounding candidate genes, including those identified in the 23 QTL regions.
(DOC)

## References

[1] von Uexküll HR, Mutert E, Date RA, Grundon NJ, Raymet GE, Probert ME (1995). Global extent, development and economic impact of acid soils.. Plant-Soil Interactions at Low pH: Principles and Management.

[2] Kochian LV, Pineros MA, Hoekenga OA (2005). The physiology, genetics and molecular biology of plant aluminum tolerance and toxicity.. Plant and Soil.

[3] Foy C (1988). Plant adaptation to acid, aluminum-toxic soils.. Communications in Soil Science and Plant Analysis.

[4] Sasaki T, Ryan P, Delhaize E, Hebb D, Ogihara Y (2006). Sequence upstream of the wheat (*Triticum aestivum* L.) *ALMT1* gene and its relationship to aluminum resistance.. Plant and Cell Physiology.

[5] Pineros M, Shaff J, Manslank H, Carvalho Alves V, Kochian L (2005). Aluminum resistance in maize cannot be solely explained by root organic acid exudation. A comparative physiological study.. Plant Physiology.

[6] Furukawa J, Yamaji N, Wang H, Mitani N, Murata Y (2007). An aluminum-activated citrate transporter in barley.. Plant and Cell Physiology.

[7] Caniato F, Guimaraes C, Schaffert R, Alves V, Kochian L (2007). Genetic diversity for aluminum tolerance in sorghum.. Theoretical and Applied Genetics.

[8] Famoso A, Clark R, Shaff J, Craft E, McCouch S (2010). Development of a novel Aluminum tolerance phenotyping platform used for comparisons of cereal Aluminum tolerance and investigations into rice Aluminum tolerance mechanisms.. Plant Physiology.

[9] Sasaki T, Yamamoto Y, Ezaki B, Katsuhara M, Ahn S (2004). A wheat gene encoding an aluminum-activated malate transporter.. The Plant Journal.

[10] Magalhaes J, Garvin D, Wang Y, Sorrells M, Klein P (2004). Comparative mapping of a major aluminum tolerance gene in sorghum and other species in the Poaceae.. Genetics.

[11] Minella E, Sorrells M (1992). Aluminum tolerance in barley: genetic relationships among genotypes of diverse origin.. Crop Science.

[12] Ninamango-Cardenas F, Teixeira Guimarães C, Martins P, Netto Parentoni S, Portilho Carneiro N (2003). Mapping QTLs for aluminum tolerance in maize.. Euphytica.

[13] Nguyen VT, Burow MD, Nguyen HT, Le BT, Le TD (2001). Molecular mapping of genes conferring aluminum tolerance in rice (*Oryza sativa* L.).. Theoretical and Applied Genetics.

[14] Sasaki T, Yamamoto Y, Ezaki E, Katsuhara M, Ryan PR (2004). A gene encoding an aluminum-activated malate transporter segregates with aluminum tolerance in wheat.. Plant Journal.

[15] Magalhaes J, Liu J, Guimarães C, Lana U, Alves V (2007). A gene in the multidrug and toxic compound extrusion (*MATE*) family confers aluminum tolerance in sorghum.. Nature Genetics.

[16] Hoekenga O, Vision T, Shaff J, Monforte A, Lee G (2003). Identification and characterization of aluminum tolerance loci in Arabidopsis (*Landsberg erecta x Columbia*) by quantitative trait locus mapping. A physiologically simple but genetically complex trait.. Plant Physiology.

[17] Nguyen V, Nguyen B, Sarkarung S, Martinez C, Paterson A (2002). Mapping of genes controlling aluminum tolerance in rice: comparison of different genetic backgrounds.. Molecular Genetics and Genomics.

[18] Huang X, Feng Q, Qian Q, Zhao Q, Wang L (2009). High-throughput genotyping by whole-genome resequencing.. Genome Research.

[19] Yamaji N, Huang C, Nagao S, Yano M, Sato Y (2009). A zinc finger transcription factor ART1 regulates multiple genes implicated in aluminum tolerance in rice.. The Plant Cell.

[20] Xia J, Yamaji N, Kasai T, Ma J (2010). Plasma membrane-localized transporter for aluminum in rice.. Proceedings of the National Academy of Sciences.

[21] Larsen P, Cancel J, Rounds M, Ochoa V (2007). Arabidopsis ALS1 encodes a root tip and stele localized half type ABC transporter required for root growth in an aluminum toxic environment.. Planta.

[22] Larsen P, Geisler M, Jones C, Williams K, Cancel J (2005). ALS3 encodes a phloem localized ABC transporter like protein that is required for aluminum tolerance in Arabidopsis.. The Plant Journal.

[23] Ma JF, Shen R, Zhao Z, Wissuwa M, Takeuchi Y (2002). Response of rice to Al stress and identification of quantitative trait Loci for Al tolerance.. Plant Cell Physiology.

[24] Huang C, Yamaji N, Mitani N, Yano M, Nagamura Y (2009). A bacterial-type ABC transporter is involved in aluminum tolerance in rice.. The Plant Cell.

[25] Nguyen BD, Brar DS, Bui BC, Nguyen TB, Pham LN (2003). Identification and mapping of the QTL for aluminum tolerance introgressed from the new source, *Oryza rufipogon* Griff., into indica rice (*Oryza sativa* L.).. Theoretical and Applied Genetics.

[26] Ma J, Shen R, Zhao Z, Wissuwa M, Takeuchi Y (2002). Response of rice to Al stress and identification of quantitative trait loci for Al tolerance.. Plant and Cell Physiology.

[27] Xue Y, Jiang L, Su N, Wang J, Deng P (2007). The genetic basic and fine-mapping of a stable quantitative-trait loci for aluminium tolerance in rice.. Planta.

[28] Xue Y, Wan J, Jiang L, Wang C, Liu L (2006). Identification of quantitative trait loci associated with aluminum tolerance in rice (*Oryza sativa* L.).. Euphytica.

[29] Wu P, Liao CD, Hu B, Yi KK, Jin WZ (2000). QTLs and epistasis for aluminum tolerance in rice (*Oryza sativa* L.) at different seedling stages.. Theoretical Applied Genetics.

[30] Oka HI, Oka HI (1988). *Indica-Japonica* differentiation of rice cultivars.. Origin of Cultivated Rice.

[31] Barbier P (1989). Genetic variation and ecotypic differentiation in the wild rice species *O. rufipogon* II. Influence of the mating system and life history traits on the genetic structure of populations.. Japanese Journal of Genetics.

[32] Ma J, Bennetzen JL (2004). Rapid recent growth and divergence of rice nuclear genomes.. Proceedings of the National Academy of Sciences.

[33] Vitte C, Ishii T, Lamy F, Brar D, Panaud O (2004). Genomic paleontology provides evidence for two distinct origins of Asian rice (*Oryza sativa* L.).. Molecular Genetics and Genomics.

[34] Londo J, Chiang Y, Hung K, Chiang T, Schaal B (2006). Phylogeography of Asian wild rice, Oryza rufipogon, reveals multiple independent domestications of cultivated rice, Oryza sativa.. Proceedings of the National Academy of Sciences.

[35] Kovach MJ, Sweeney MT, McCouch SR (2007). New insights into the history of rice domestication.. Trends in Genetics.

[36] Kovach M, McCouch S (2008). Leveraging natural diversity: back through the bottleneck.. Current Opinion in Plant Biology.

[37] Zhu Q, Ge S (2005). Phylogenetic relationships among A-genome species of the genus *Oryza* revealed by intron sequences of four nuclear genes.. New Phytologist.

[38] Zhou H, Xie Z, Ge S (2003). Microsatellite analysis of genetic diversity and population genetic structure of a wild rice (*Oryza rufipogon* Griff.) in China.. Theoretical and Applied Genetics.

[39] Sweeney M, Thomson M, Cho Y-G, Park Y-J, Williamson S (2007). Global dissemination of a single mutation conferring white pericarp in rice.. PLoS Genet.

[40] Garris AJ, Tai TH, Coburn JR, Kresovich S, McCouch S (2005). Genetic structure and diversity in Oryza sativa L.. Genetics.

[41] Caicedo AL, Williamson SH, Hernandez RD, Boyko A, Fledel-Alon A (2007). Genome-wide patterns of nucleotide polymorphism in domesticated rice.. PLoS Genet.

[42] Zhao K, Wright M, Kimball J, Eizenga G, McClung A (2010). Genomic diversity and introgression in O. sativa reveal the impact of domestication and breeding on the rice genome.. PLoS ONE.

[43] Khush G (1997). Origin, dispersal, cultivation and variation of rice.. Plant Molecular Biology.

[44] Glaszmann JC (1987). Isozymes and classification of Asian rice varieties.. Theoretical and Applied Genetics.

[45] Garris AJ, McCouch SR, Kresovich S (2003). Population structure and its effect on haplotype diversity and linkage disequilibrium surrounding the xa5 locus of rice (*Oryza sativa* L.).. Genetics.

[46] Olsen K, Caicedo A, Polato N, McClung A, McCouch S (2006). Selection under domestication: evidence for a sweep in the rice Waxy genomic region.. Genetics.

[47] Mather K, Caicedo A, Polato N, Olsen K, McCouch S (2007). The extent of linkage disequilibrium in rice (*Oryza sativa* L.).. Genetics.

[48] Rakshit S, Rakshit A, Matsumura H, Takahashi Y, Hasegawa Y (2007). Large-scale DNA polymorphism study of *Oryza sativa* and *O. rufipogon* reveals the origin and divergence of Asian rice.. Theoretical and Applied Genetics.

[49] McNally KL, Childs KL, Bohnert R, Davidson RM, Zhao K (2009). Genomewide SNP variation reveals relationships among landraces and modern varieties of rice.. Proceedings of the National Academy of Sciences.

[50] Reich D, Cargill M, Bolk S, Ireland J, Sabeti P (2001). Linkage disequilibrium in the human genome.. Nature.

[51] Daly M, Rioux J, Schaffner S, Hudson T, Lander E (2001). High-resolution haplotype structure in the human genome.. Nature Genetics.

[52] Jeffreys A, Kauppi L, Neumann R (2001). Intensely punctate meiotic recombination in the class II region of the major histocompatibility complex.. Nature Genetics.

[53] Kim S, Plagnol V, Hu TT, Toomajian C, Clark RM (2007). Recombination and linkage disequilibrium in *Arabidopsis thaliana*.. Nature Genetics.

[54] Nordborg M, Borevitz JO, Bergelson J, Berry CC, Chory J (2002). The extent of linkage disequilibrium in Arabidopsis thaliana.. Nature Genetics.

[55] Jung M, Ching A, Bhattramakki D, Dolan M, Tingey S (2004). Linkage disequilibrium and sequence diversity in a 500-kbp region around the adh1 locus in elite maize germplasm.. Theoretical and Applied Genetics.

[56] Yu J, Buckler ES (2006). Genetic association mapping and genome organization of maize.. Current Opinion in Biotechnology.

[57] Ching A, Caldwell K, Jung M, Dolan M, Smith O (2002). SNP frequency, haplotype structure and linkage disequilibrium in elite maize inbred lines.. BMC Genetics.

[58] Tenaillon M, Sawkins M, Long A, Gaut R, Doebley J (2001). Patterns of DNA sequence polymorphism along chromosome 1 of maize (Zea mays ssp. mays L.).. Proceedings of the National Academy of Sciences of the United States of America.

[59] Remington D, Thornsberry J, Matsuoka Y, Wilson L, Whitt S (2001). Structure of linkage disequilibrium and phenotypic associations in the maize genome.. Proceedings of the National Academy of Sciences of the United States of America.

[60] Platt A, Horton M, Huang Y, Li Y, Anastasio A (2010). The scale of population structure in Arabidopsis thaliana.. PLoS Genet.

[61] Atwell S, Huang YS, Vilhjalmsson BJ, Willems G, Horton M (2010). Genome-wide association study of 107 phenotypes in Arabidopsis thaliana inbred lines.. Nature.

[62] Zhao K, Aranzana M, Kim S, Lister C, Shindo C (2007). An Arabidopsis example of association mapping in structured samples.. PLoS Genet.

[63] Yu J, Pressoir G, Briggs W, Bi I, Yamasaki M (2005). A unified mixed-model method for association mapping that accounts for multiple levels of relatedness.. Nature Genetics.

[64] Huang X, Wei X, Sang T, Zhao Q, Feng Q (2010). Genome-wide association studies of 14 agronomic traits in rice landraces.. Nature Genetics.

[65] Zhao KT, CW, Eizenga GC, Wright MH, Ali ML, Price AH, Norton GJ, Islam MR, Reynolds A, Mezey J, McClung AM, Bustamante CD, McCouch SR (2011). Genome-wide association mapping reveals rich genetic architecture of complex traits in *Oryza sativa*.. Nature Communications (Accepted).

[66] Ali M, McClung AM, Jia MH, Kimball JA, McCouch SR, Eizenga GC (2011). Rice Diversity Panel “evaluated for genetic and agro-morphological diversity between subpopulations and its geographic distribution”.. Crop Science.

[67] Tung C-W, Zhao K, Wright M, Ali M, Jung J (2010). Development of a research platform for dissecting phenotype–genotype associations in rice (*Oryza* spp.).. Rice.

[68] McCouch SR, Zhao K, Wright M, Tung C-W, Ebana K (2010). Development of genome-wide SNP assays for rice.. Breeding Science.

[69] Ahmadi N, Dubreuil-Tranchant C, Courtois B, Fonceka D, This D, IRRI, editor New resources and integrated maps for IR64 × Azucena: a reference population in rice.. 2005 19–23 November 2005.

[70] Lin SY, Sasaki T, Yano M (1998). Mapping quantitative trait loci controlling seed dormancy and heading date in rice, *Oryza sativa* L., using backcross inbred lines.. Theoretical and Applied Genetics.

[71] Kang H, Zaitlen N, Wade C, Kirby A, Heckerman D (2008). Efficient control of population structure in model organism association mapping.. Genetics.

[72] Li Y, Huang Y, Bergelson J, Nordborg M, Borevitz JO (2010). Association mapping of local climate-sensitive quantitative trait loci in Arabidopsis thaliana.. Proceedings of the National Academy of Sciences.

[73] Maron LG, Kirst M, Mao C, Milner MJ, Menossi M (2008). Transcriptional profiling of aluminum toxicity and tolerance responses in maize roots.. New Phytologist.

[74] Krill A, Kirst M, Kochian L, Buckler E, Hoekenga O (2010). Association and linkage analysis of Aluminum tolerance genes in maize.. PLoS ONE.

[75] Sang T, Ge S (2007). The puzzle of rice domestication.. Journal of Integrative Plant Biology.

[76] Han B, Xue Y (2003). Genome-wide intraspecific DNA-sequence variations in rice.. Current Opinion in Plant Biology.

[77] Liu X, Lin F, Wang L, Pan Q (2007). The in silico map-based cloning of Pi36, a rice coiled-coil–nucleotide-binding site–leucine-rich repeat gene that confers race-specific resistance to the blast fungus.. Genetics.

[78] Kumagai M, Wang L, Ueda S (2010). Genetic diversity and evolutionary relationships in genus *Oryza* revealed by using highly variable regions of chloroplast DNA.. Gene.

[79] Beavis WD (1994). The power and deceit of QTL experiments: lessons from comparative QTL studies..

[80] Baier AC, Somers DJ, Gusiafson JP (1995). Aluminium tolerance in wheat: correlating hydroponic evaluations with field and soil performances.. Plant Breeding.

[81] Basten C, Weir B, Zeng Z (2002). QTL Cartographer, version 1.16..

[82] Price A, Patterson N, Plenge R, Weinblatt M, Shadick N (2006). Principal components analysis corrects for stratification in genome-wide association studies.. Nature Genetics.

[83] Barrett JC, Fry B, Maller J, Daly MJ (2005). Haploview: analysis and visualization of LD and haplotype maps.. Bioinformatics.

[84] Pritchard J, Stephens M, Donnelly P (2000). Inference of population structure using multilocus genotype data.. Genetics.

