# Supplementary material for: Genetic Architecture of Aluminum Tolerance in Rice (Oryza sativa) Determined through Genome-Wide Association Analysis and QTL Mapping
Source: PLoS Genet. 2011 Aug 4;7(8):e1002221. doi: 10.1371/journal.pgen.1002221 (PMC3150440; doi:10.1371/journal.pgen.1002221)
Supplement: Table S1 — Aluminum tolerance and subpopulation identity of 383 genotypes from the rice diversity panel. Ten genotypes denoted with asterisk (*), did not have existing SNP genotype data at the time of GWA analysis and were not included in the GWA analysis. Subpopulation ancestry was based off 80% identity: AUS = aus; IND = indica; TRJ = tropical japonica; TEJ = temperate japonica; Group V is also known as aromatic. Any line with less than 80% subpopulation identity was considered an admixture (ADMIX). The two major varietal groups are Indica and Japonica; the Indica varietal group is comprised of the aus and indica subpopulations and the Japonica varietal group is comprised of the temperate japonica, tropical japonica, and group V subpopulations. (DOC) [file pgen.1002221.s006.doc]

**Table S1.**

| **Line I.D. (NSF-TV)** | **GSOR I.D.** | **TRG-RRG** | **Accession Name** | **Subpopulation (80% identity)** | **Varietal Group (80% identity)** |
| --- | --- | --- | --- | --- | --- |
|
| 1 | 301001 | 0.730 | Agostano | TEJ | *Japonica* |
| 2 | 301002 | 0.784 | *AICHI ASAHI | TEJ | *Japonica* |
| 3 | 301003 | 0.300 | Ai-Chiao-Hong | IND | *Indica* |
| 4 | 301004 | 0.540 | Arc 10177 | AUS | *Indica* |
| 5 | 301005 | 0.440 | Arc 10352 | Group V | *Japonica* |
| 6 | 301006 | 0.570 | Arc 7229 | AUS | *Indica* |
| 7 | 301007 | 0.860 | Arias | TRJ | *Japonica* |
| 8 | 301008 | 0.664 | Asse Y Pung | TRJ | *Japonica* |
| 9 | 301009 | 0.723 | Baber | TEJ | *Japonica* |
| 10 | 301010 | 0.902 | Baghlani Nangarhar | TEJ | *Japonica* |
| 12 | 301011 | 0.130 | Basmati | Group V | *Japonica* |
| 13 | 301012 | 0.390 | Basmati 1 | AUS | *Indica* |
| 14 | 301013 | 0.440 | Basmati 217 | TRJ | *Japonica* |
| 15 | 301383 | 0.829 | Beonjo | TEJ | *Japonica* |
| 16 | 301014 | 0.680 | Bico Branco | Group V | *Japonica* |
| 17 | 301015 | 0.460 | Binulawan | IND | *Indica* |
| 18 | 301016 | 0.420 | Bj 1 | AUS | *Indica* |
| 19 | 301017 | 0.410 | Black Gora | AUS | *Indica* |
| 20 | 301018 | 0.550 | Blue Rose | ADMIX | *Japonica* |
| 21 | 301019 | 0.310 | Byakkoku Y 5006 Seln | IND | *Indica* |
| 22 | 301020 | 0.654 | Caawa/Fortuna 6-103-15 | TRJ | *Japonica* |
| 23 | 301021 | 0.460 | Canella De Ferro | TRJ | *Japonica* |
| 24 | 301022 | 0.690 | Carolina Gold | TRJ | *Japonica* |
| 25 | 301023 | 0.736 | Carolina Gold | TRJ | *Japonica* |
| 26 | 301024 | 0.562 | Carolina Gold Sel | TRJ | *Japonica* |
| 27 | 301025 | 0.490 | Chahora 144 | TRJ | *Japonica* |
| 28 | 301026 | 0.250 | *CHAMPA TONG 54 | AUS | *Indica* |
| 30 | 301028 | 0.760 | Chiem Chanh | IND | *Indica* |
| 31 | 301029 | 0.780 | Chinese | TEJ | *Japonica* |
| 32 | 301030 | 1.050 | Chodongji | TEJ | *Japonica* |
| 33 | 301031 | 0.240 | Q33 | AUS | *Indica* |
| 35 | 301033 | 0.451 | Co18 | IND | *Indica* |
| 36 | 301034 | 0.730 | CS-M3 | TEJ | *Japonica* |
| 37 | 301035 | 0.470 | Cuba 65 | TRJ | *Japonica* |
| 38 | 301036 | 0.640 | *DA 5 | IND | *Indica* |
| 39 | 301037 | 0.287 | Da16 | ADMIX | *Indica* |
| 40 | 301038 | 0.779 | Dam | ADMIX | *Japonica* |
| 43 | 301040 | 0.320 | Dee Geo Woo Gen | IND | *Indica* |
| 44 | 301041 | 0.440 | Dhala Shaitta | AUS | *Indica* |
| 45 | 301042 | 0.600 | Dom-Sofid | Group V | *Japonica* |
| 46 | 301043 | 0.650 | Dourado Agulha | TRJ | *Japonica* |
| 48 | 301044 | 0.642 | *DULAR | AUS | *Indica* |
| 49 | 301045 | 0.350 | Dv85 | AUS | *Indica* |
| 50 | 301046 | 0.120 | Dz78 | AUS | *Indica* |
| 51 | 301047 | 0.600 | Early Wataribune | TEJ | *Japonica* |
| 52 | 301048 | 0.180 | Eh Ia Chiu | TEJ | *Japonica* |
| 53 | 301049 | 0.590 | Firooz | Group V | *Japonica* |
| 54 | 301050 | 0.680 | Fortuna | TRJ | *Japonica* |
| 55 | 301051 | 0.740 | Gerdeh | ADMIX | *Japonica* |
| 56 | 301052 | 0.810 | Geumobyeo | TEJ | *Japonica* |
| 57 | 301053 | 0.300 | Gharib | IND | *Indica* |
| 58 | 301054 | 0.309 | Ghati Kamma Nangarhar | AUS | *Indica* |
| 59 | 301055 | 0.944 | Gogo Lempuk | TRJ | *Japonica* |
| 60 | 301056 | 0.600 | Gotak Gatik | ADMIX | *Japonica* |
| 61 | 301057 | 0.430 | Guan-Yin-Tsan | IND | *Indica* |
| 62 | 301386 | 0.617 | Gyehwa 3 | TEJ | *Japonica* |
| 63 | 301387 | 0.563 | Haginomae Mochi | TEJ | *Japonica* |
| 64 | 301388 | 0.684 | Heukgyeong | TEJ | *Japonica* |
| 65 | 301058 | 0.530 | Honduras | TRJ | *Japonica* |
| 66 | 301059 | 1.000 | 66 | IND | *Indica* |
| 67 | 301060 | 0.800 | Hu Lo Tao | TEJ | *Japonica* |
| 68 | 301061 | 0.646 | 68 | ADMIX | *Admixed* |
| 69 | 301062 | 0.690 | Iac 25 | TRJ | *Japonica* |
| 70 | 301063 | 0.630 | Iguape Cateto | TRJ | *Japonica* |
| 71 | 301064 | 0.370 | Ir 36 | IND | *Indica* |
| 72 | 301065 | 0.280 | Ir 8 | IND | *Indica* |
| 73 | 301066 | 0.660 | Irat 177 | TRJ | *Japonica* |
| 74 | 301067 | 0.320 | Irga 409 | IND | *Indica* |
| 75 | 301068 | 0.650 | Jambu | TRJ | *Admixed* |
| 76 | 301069 | 0.280 | Jaya | IND | *Indica* |
| 77 | 301070 | 0.310 | Jc149 | IND | *Indica* |
| 78 | 301071 | 0.410 | Jhona 349 | AUS | *Indica* |
| 79 | 301072 | 0.815 | Jouiku 393G | TEJ | *Japonica* |
| 80 | 301073 | 0.756 | K 65 | ADMIX | *Admixed* |
| 81 | 301074 | 0.480 | Kalamkati | AUS | *Indica* |
| 82 | 301389 | 0.440 | *KALUKANTHA | IND | *Indica* |
| 83 | 301075 | 0.620 | Kamenoo | TEJ | *Japonica* |
| 84 | 301076 | 0.774 | Kaniranga | TRJ | *Japonica* |
| 85 | 301077 | 0.201 | Kasalath | AUS | *Indica* |
| 87 | 301079 | 0.663 | Keriting Tingii | ADMIX | *Japonica* |
| 88 | 301080 | 0.591 | Khao Gaew | AUS | *Indica* |
| 89 | 301081 | 0.600 | Khao Hawm | TRJ | *Japonica* |
| 90 | 301082 | 0.190 | Kiang-Chou-Chiu | IND | *Indica* |
| 91 | 301083 | 0.932 | Kibi | TEJ | *Japonica* |
| 92 | 301084 | 0.590 | Kinastano | TRJ | *Japonica* |
| 93 | 301085 | 0.580 | Kitrana 508 | Group V | *Japonica* |
| 94 | 301086 | 0.490 | Koshihikari | TEJ | *Japonica* |
| 96 | 301088 | 0.604 | Ku115 | ADMIX | *Japonica* |
| 97 | 301089 | 0.448 | Kun-Min-Tsieh-Hunan | IND | *Indica* |
| 98 | 301090 | 0.689 | L-202 | TRJ | *Japonica* |
| 99 | 301091 | 0.580 | Lac 23 | TRJ | *Japonica* |
| 100 | 301092 | 0.800 | Lacrosse | ADMIX | *Japonica* |
| 101 | 301093 | 0.630 | Lemont | TRJ | *Japonica* |
| 102 | 301094 | 0.440 | 102 | IND | *Indica* |
| 103 | 301095 | 0.550 | Luk Takhar | TEJ | *Japonica* |
| 104 | 301096 | 0.780 | Mansaku | TEJ | *Japonica* |
| 105 | 301097 | 0.400 | Mehr | AUS | *Indica* |
| 106 | 301098 | 0.450 | Ming Hui | IND | *Indica* |
| 107 | 301099 | 1.180 | Miriti | TRJ | *Japonica* |
| 108 | 301100 | 1.317 | Moroberekan | TRJ | *Japonica* |
| 109 | 301101 | 0.333 | Mtu9 | IND | *Indica* |
| 110 | 301102 | 0.290 | Mudgo | IND | *Indica* |
| 111 | 301104 | 0.510 | Q32-111 | TRJ | *Japonica* |
| 112 | 301104 | 0.150 | N12 | Group V | *Japonica* |
| 113 | 301105 | 0.640 | Norin 20 | TEJ | *Japonica* |
| 114 | 301106 | 0.510 | Nova | ADMIX | *Japonica* |
| 116 | 301108 | 0.700 | Npe 844 | TRJ | *Japonica* |
| 117 | 301109 | 0.320 | O-Luen-Cheung | IND | *Indica* |
| 118 | 301110 | 0.530 | Oro | TEJ | *Japonica* |
| 119 | 301111 | 0.300 | Oryzica Llanos 5 | IND | *Admixed* |
| 120 | 301112 | 0.450 | Os6 | TRJ | *Japonica* |
| 121 | 301113 | 0.830 | Ostiglia | TEJ | *Japonica* |
| 122 | 301114 | 0.692 | Padi Kasalle | TRJ | *Japonica* |
| 123 | 301115 | 0.320 | Pagaiyahan | IND | *Indica* |
| 125 | 301116 | 0.260 | Pao-Tou-Hung | IND | *Indica* |
| 126 | 301117 | 0.578 | Pappaku | IND | *Indica* |
| 127 | 301118 | 0.430 | *PATNAI 23 | IND | *Indica* |
| 128 | 301119 | 0.823 | Pato De Gallinazo | ADMIX | *Japonica* |
| 129 | 301120 | 0.300 | Peh-Kuh | IND | *Indica* |
| 130 | 301121 | 0.340 | Peh-Kuh-Tsao-Tu | IND | *Indica* |
| 131 | 301122 | 0.160 | Phudugey | AUS | *Indica* |
| 132 | 301123 | 0.350 | Rathuwee | IND | *Indica* |
| 133 | 301124 | 1.000 | Rikuto Kemochi | TEJ | *Japonica* |
| 134 | 301125 | 0.480 | Romeo | TEJ | *Japonica* |
| 135 | 301126 | 0.698 | RT 1031-69 | TRJ | *Japonica* |
| 137 | 301128 | 0.250 | Rts14 | IND | *Indica* |
| 138 | 301129 | 0.580 | Rts4 | IND | *Indica* |
| 139 | 301130 | 0.690 | S4542A3-49B-2B12 | TRJ | *Japonica* |
| 140 | 301131 | 0.740 | Saturn | ADMIX | *Japonica* |
| 141 | 301132 | 0.250 | Seratoes Hari | IND | *Indica* |
| 142 | 301133 | 1.150 | Shai-Kuh | IND | *Indica* |
| 143 | 301134 | 0.810 | Shinriki | TEJ | *Japonica* |
| 145 | 301136 | 0.030 | Short Grain | IND | *Indica* |
| 147 | 301138 | 0.580 | Sinampaga Selection | TRJ | *Japonica* |
| 148 | 301139 | 0.360 | Sintane Diofor | IND | *Indica* |
| 149 | 301140 | 0.920 | Sinaguing | TRJ | *Japonica* |
| 150 | 301141 | 0.530 | Sultani | TRJ | *Japonica* |
| 151 | 301142 | 0.730 | Suweon | TEJ | *Japonica* |
| 152 | 301143 | 0.246 | T 1 | AUS | *Indica* |
| 153 | 301144 | 0.260 | T26 | AUS | *Indica* |
| 154 | 301145 | 0.670 | Ta Hung Ku | TEJ | *Japonica* |
| 155 | 301146 | 0.750 | Ta Mao Tsao | TEJ | *Japonica* |
| 156 | 301147 | 0.290 | Taichung Native 1 | IND | *Indica* |
| 157 | 301148 | 0.540 | Tainan Iku 487 | TEJ | *Japonica* |
| 158 | 301149 | 0.710 | Taipei 309 | TEJ | *Japonica* |
| 159 | 301150 | 0.555 | Tam Cau 9A | IND | *Indica* |
| 160 | 301151 | 0.500 | Tchampa | Group V | *Japonica* |
| 161 | 301152 | 0.320 | Teqing | IND | *Indica* |
| 162 | 301153 | 0.434 | Tkm6 | IND | *Indica* |
| 163 | 301154 | 0.800 | Taducan | IND | *Indica* |
| 164 | 301155 | 0.706 | Tondok | TRJ | *Japonica* |
| 165 | 301156 | 0.700 | Trembese | TRJ | *Japonica* |
| 166 | 301157 | 0.410 | Tsipala 421 | ADMIX | *Indica* |
| 167 | 301158 | 0.570 | B6616A4-22-Bk-5-4 | TRJ | *Japonica* |
| 168 | 301159 | 0.240 | Vary Vato 462 | ADMIX | *Indica* |
| 169 | 301160 | 0.750 | WC 6 | TEJ | *Japonica* |
| 170 | 301161 | 0.910 | Wells | TRJ | *Japonica* |
| 171 | 301162 | 0.280 | Zhe 733 | IND | *Indica* |
| 172 | 301163 | 0.240 | Zhenshan 2 | IND | *Indica* |
| 173 | 301164 | 0.750 | Nipponbare | TEJ | *Japonica* |
| 174 | 301165 | 0.768 | Azucena | TRJ | *Japonica* |
| 175 | 301167 | 0.785 | 1021 | TRJ | *Japonica* |
| 176 | 301167 | 0.779 | 583 | TRJ | *Japonica* |
| 177 | 301168 | 0.926 | 68-2 | TEJ | *Japonica* |
| 178 | 301169 | 0.180 | Arc 6578 | AUS | *Indica* |
| 179 | 301170 | 0.850 | Bellardone | TEJ | *Japonica* |
| 180 | 301171 | 0.986 | Benllok | TEJ | *Japonica* |
| 181 | 301172 | 0.470 | Bergreis | TEJ | *Japonica* |
| 182 | 301173 | 0.788 | Blue Rose Supreme | ADMIX | *Japonica* |
| 183 | 301174 | 0.713 | Boa Vista | TRJ | *Japonica* |
| 184 | 301175 | 0.911 | Bombon | TEJ | *Japonica* |
| 185 | 301176 | 0.768 | 185 | TRJ | *Japonica* |
| 186 | 301177 | 1.030 | Bul Zo | TEJ | *Japonica* |
| 187 | 301178 | 0.549 | C57-5043 | TRJ | *Japonica* |
| 188 | 301179 | 0.530 | Coppocina | TRJ | *Japonica* |
| 189 | 301180 | 0.440 | Criollo La Fria | IND | *Indica* |
| 190 | 301181 | 0.680 | Delrex | TRJ | *Japonica* |
| 191 | 301182 | 0.643 | Dom Zard | Group V | *Japonica* |
| 192 | 301183 | 0.750 | Erythroceros Hokkaido | TEJ | *Japonica* |
| 193 | 301184 | 0.641 | Fossa Av | TRJ | *Japonica* |
| 195 | 301186 | 0.704 | Irat 13 | TRJ | *Japonica* |
| 196 | 301187 | 0.377 | Jm70 | IND | *Indica* |
| 197 | 301188 | 0.760 | Kaukkyi Ani | ADMIX | *Japonica* |
| 198 | 301189 | 0.797 | Leah | TRJ | *Japonica* |
| 199 | 301190 | 0.873 | Mojito Colorado | TRJ | *Japonica* |
| 200 | 301191 | 0.441 | P 737 | AUS | *Indica* |
| 201 | 301192 | 0.676 | Pate Blanc Mn 1 | TRJ | *Japonica* |
| 202 | 301193 | 0.590 | Pratao | TRJ | *Japonica* |
| 203 | 301194 | 0.270 | Radin Ebos 33 | IND | *Indica* |
| 204 | 301195 | 0.617 | Razza 77 | TEJ | *Japonica* |
| 205 | 301196 | 0.779 | Rinaldo Bersani | ADMIX | *Japonica* |
| 206 | 301197 | 0.350 | Rojofotsy 738 | ADMIX | *Indica* |
| 207 | 301198 | 0.469 | Sigadis | IND | *Indica* |
| 208 | 301199 | 0.590 | Slo 17 | IND | *Indica* |
| 209 | 301200 | 0.250 | Tchibanga | IND | *Indica* |
| 211 | 301202 | 0.875 | Tokyo Shino Mochi | ADMIX | *Japonica* |
| 212 | 301204 | 0.765 | Wc 2810 | TRJ | *Japonica* |
| 213 | 301204 | 0.721 | Wc 3397 | TRJ | *Japonica* |
| 214 | 301205 | 0.650 | Wc 4419 | TRJ | *Japonica* |
| 215 | 301206 | 0.750 | Wc 4443 | TRJ | *Japonica* |
| 216 | 301207 | 0.790 | Yabani Montakhab 7 | TEJ | *Japonica* |
| 217 | 301208 | 0.690 | Yrl-1 | ADMIX | *Japonica* |
| 218 | 301209 | 0.960 | Pi 298967-1 | ADMIX | *Japonica* |
| 219 | 301210 | 0.809 | Nucleoryza | TEJ | *Japonica* |
| 221 | 301212 | 0.680 | Sadri Belyi | Group V | *Japonica* |
| 222 | 301213 | 0.350 | Paraiba Chines Nova | IND | *Indica* |
| 223 | 301214 | 0.614 | Priano Guaira | TRJ | *Japonica* |
| 224 | 301215 | 0.620 | Karabaschak | TEJ | *Japonica* |
| 225 | 301216 | 0.866 | Biser 1 | TEJ | *Japonica* |
| 226 | 301217 | 0.853 | Irat 44 | TRJ | *Japonica* |
| 227 | 301218 | 0.250 | Riz Local | ADMIX | *Indica* |
| 228 | 301219 | 0.400 | Ca 902/B/2/1 | AUS | *Indica* |
| 229 | 301220 | 0.560 | Niquen | TRJ | *Japonica* |
| 231 | 301221 | 0.427 | Hunan Early Dwarf No. 3 | IND | *Indica* |
| 232 | 301222 | 0.997 | Shangyu 394 | TEJ | *Japonica* |
| 233 | 301223 | 0.894 | Sung Liao 2 | TEJ | *Japonica* |
| 234 | 301224 | 0.310 | Aijiaonante | IND | *Indica* |
| 235 | 301225 | 0.290 | Sze Guen Zim | IND | *Indica* |
| 236 | 301226 | 0.713 | Wc 521 | ADMIX | *Japonica* |
| 237 | 301227 | 0.530 | Estrela | ADMIX | *Japonica* |
| 238 | 301228 | 0.720 | *WAB56-104 | TRJ | *Japonica* |
| 239 | 301229 | 0.731 | WAB 502-13-4-1 | TRJ | *Japonica* |
| 240 | 301230 | 0.607 | Wab 501-11-5-1 | TRJ | *Japonica* |
| 241 | 301231 | 0.170 | Ecia76-S89-1 | IND | *Indica* |
| 242 | 301232 | 0.603 | 27 | TRJ | *Japonica* |
| 243 | 301233 | 1.080 | Tropical Rice | TEJ | *Japonica* |
| 244 | 301234 | 0.582 | Arabi | ADMIX | *Japonica* |
| 245 | 301235 | 0.729 | Sab Ini | TEJ | *Japonica* |
| 246 | 301236 | 0.360 | Saraya | AUS | *Indica* |
| 247 | 301237 | 0.661 | Desvauxii | TEJ | *Japonica* |
| 248 | 301238 | 0.600 | Caucasica | TEJ | *Japonica* |
| 249 | 301239 | 0.909 | Pirinae 69 | ADMIX | *Admixed* |
| 250 | 301240 | 0.833 | Bulgare | TEJ | *Japonica* |
| 251 | 301241 | 0.530 | H256-76-1-1-1 | TRJ | *Japonica* |
| 252 | 301242 | 0.430 | Djimoron | IND | *Indica* |
| 253 | 301243 | 0.575 | Guineandao | ADMIX | *Japonica* |
| 254 | 301244 | 0.400 | Hon Chim | IND | *Indica* |
| 255 | 301245 | 0.370 | Pai Hok Glutinous | IND | *Indica* |
| 256 | 301246 | 0.510 | Romanica | TEJ | *Japonica* |
| 257 | 301247 | 1.026 | Agusita | TEJ | *Japonica* |
| 258 | 301248 | 0.730 | Tia Bura | TRJ | *Japonica* |
| 259 | 301249 | 0.300 | Sadri Tor Misri | ADMIX | *Indica* |
| 260 | 301250 | 0.620 | NSF-TV 260 | Group V | *Japonica* |
| 261 | 301251 | 0.190 | Shim Balte | AUS | *Indica* |
| 262 | 301252 | 0.470 | Halwa Gose Red | AUS | *Indica* |
| 263 | 301253 | 0.660 | Maratelli | TEJ | *Japonica* |
| 264 | 301254 | 0.552 | Baldo | ADMIX | *Japonica* |
| 265 | 301255 | 0.910 | Vialone | TEJ | *Japonica* |
| 266 | 301256 | 0.717 | Hiderisirazu | ADMIX | *Japonica* |
| 267 | 301257 | 0.828 | Hatsunishiki | TEJ | *Japonica* |
| 268 | 301258 | 0.670 | Vavilovi | TEJ | *Japonica* |
| 269 | 301259 | 0.430 | Sundensis | IND | *Admixed* |
| 270 | 301260 | 0.664 | Osogovka | ADMIX | *Japonica* |
| 271 | 301261 | 0.798 | M. Blatec | ADMIX | *Japonica* |
| 272 | 301262 | 0.517 | 923 | ADMIX | *Admixed* |
| 273 | 301263 | 0.752 | Varyla | ADMIX | *Japonica* |
| 274 | 301264 | 0.660 | Padi Pagalong | TRJ | *Japonica* |
| 275 | 301265 | 0.723 | Sri Malaysia Dua | TEJ | *Japonica* |
| 276 | 301266 | 0.400 | Kaukau | AUS | *Indica* |
| 277 | 301267 | 0.690 | Gambiaka Sebela | TEJ | *Japonica* |
| 278 | 301268 | 0.513 | C1-6-5-3 | ADMIX | *Admixed* |
| 279 | 301269 | 0.602 | Kon Suito | TEJ | *Japonica* |
| 280 | 301270 | 0.744 | Saku | ADMIX | *Japonica* |
| 281 | 301271 | 0.850 | Patna | TEJ | *Japonica* |
| 282 | 301272 | 0.726 | Triomphe Du Maroc | TEJ | *Japonica* |
| 283 | 301273 | 0.700 | Chibica | TEJ | *Japonica* |
| 284 | 301274 | 0.390 | IR-44595 | IND | *Indica* |
| 285 | 301275 | 0.480 | Tox 782-20-1 | TRJ | *Japonica* |
| 286 | 301276 | 0.575 | Iita 135 | TRJ | *Japonica* |
| 287 | 301277 | 0.845 | Zerawchanica Karatalski | TEJ | *Japonica* |
| 288 | 301278 | 0.695 | Italica Carolina | TEJ | *Japonica* |
| 289 | 301279 | 1.110 | Lusitano | TEJ | *Japonica* |
| 290 | 301280 | 1.100 | Amposta | TEJ | *Japonica* |
| 291 | 301281 | 0.682 | Toploea 70/76 | TEJ | *Japonica* |
| 292 | 301282 | 0.842 | Stegaru 65 | TEJ | *Japonica* |
| 293 | 301283 | 0.160 | Tog 7178 | ADMIX | *Indica* |
| 294 | 301284 | 0.440 | SL 22-613 | ADMIX | *Admixed* |
| 295 | 301285 | 0.639 | Bombilla | TEJ | *Japonica* |
| 296 | 301286 | 1.000 | Dosel | TEJ | *Japonica* |
| 297 | 301287 | 0.823 | Bahia | TEJ | *Japonica* |
| 298 | 301288 | 0.310 | Ld 24 | IND | *Indica* |
| 299 | 301289 | 0.260 | Sml 242 | IND | *Indica* |
| 300 | 301290 | 1.066 | Sml Kapuri | TEJ | *Japonica* |
| 301 | 301291 | 0.744 | Melanotrix | TEJ | *Japonica* |
| 302 | 301292 | 0.662 | Wir 3039 | TEJ | *Japonica* |
| 303 | 301293 | 0.896 | Kihogo | TEJ | *Japonica* |
| 304 | 301294 | 0.140 | 519 | IND | *Indica* |
| 305 | 301295 | 0.632 | Doble Carolina Rinaldo Barsani | ADMIX | *Japonica* |
| 306 | 301296 | 0.849 | Wir 3764 | TEJ | *Japonica* |
| 307 | 301297 | 0.881 | Uzbekskij 2 | TEJ | *Japonica* |
| 308 | 301298 | 0.649 | Llanero 501 | TRJ | *Japonica* |
| 309 | 301299 | 0.716 | Manzano | TRJ | *Japonica* |
| 310 | 301300 | 0.611 | R 101 | TRJ | *Japonica* |
| 311 | 301301 | 0.823 | 56-122-23 | TEJ | *Japonica* |
| 312 | 301302 | 0.390 | Aswina 330 | AUS | *Indica* |
| 313 | 301303 | 0.410 | Br24 | IND | *Indica* |
| 314 | 301304 | 0.180 | Ctg 1516 | AUS | *Indica* |
| 315 | 301305 | 0.330 | Dawebyan | IND | *Indica* |
| 316 | 301306 | 0.490 | Dd 62 | AUS | *Indica* |
| 317 | 301307 | 0.080 | Dj 123 | AUS | *Indica* |
| 318 | 301308 | 0.390 | Dj 24 | AUS | *Indica* |
| 319 | 301309 | 0.280 | DK 12 | AUS | *Indica* |
| 320 | 301310 | 0.630 | Dm 43 | AUS | *Indica* |
| 321 | 301311 | 0.440 | Dm 56 | AUS | *Indica* |
| 322 | 301312 | 0.540 | DM 59 | AUS | *Indica* |
| 323 | 301313 | 0.410 | Dnj 140 | AUS | *Indica* |
| 324 | 301314 | 0.350 | Dv 123 | AUS | *Indica* |
| 325 | 301315 | 0.220 | Emata A 16-34 | IND | *Indica* |
| 326 | 301316 | 0.130 | Ghorbhai | AUS | *Indica* |
| 327 | 301317 | 0.620 | Goria | AUS | *Indica* |
| 328 | 301318 | 0.310 | Jamir | AUS | *Indica* |
| 329 | 301319 | 0.340 | Kachilon | AUS | *Indica* |
| 330 | 301320 | 0.300 | Khao Pahk Maw | AUS | *Indica* |
| 331 | 301321 | 0.340 | Khao Tot Long 227 | AUS | *Indica* |
| 332 | 301322 | 0.350 | Kpf-16 | ADMIX | *Indica* |
| 333 | 301323 | 0.656 | Leuang Hawn | TEJ | *Japonica* |
| 334 | 301324 | 1.107 | Lomello | TEJ | *Japonica* |
| 335 | 301325 | 0.550 | Okshitmayin | ADMIX | *Japonica* |
| 336 | 301326 | 0.270 | Paung Malaung | AUS | *Indica* |
| 337 | 301327 | 0.780 | Sabharaj | IND | *Indica* |
| 338 | 301328 | 0.850 | Sitpwa | TEJ | *Japonica* |
| 339 | 301329 | 0.430 | Yodanya | IND | *Indica* |
| 340 | 301330 | 0.350 | Berenj | ADMIX | *Admixed* |
| 341 | 301331 | 0.320 | Shirkati | AUS | *Indica* |
| 342 | 301332 | 0.622 | Cenit | TRJ | *Japonica* |
| 343 | 301333 | 0.712 | Victoria F.A. | ADMIX | *Japonica* |
| 344 | 301334 | 0.350 | Habiganj Boro 6 | ADMIX | *Admixed* |
| 345 | 301335 | 0.140 | Dz 193 | AUS | *Indica* |
| 346 | 301336 | 0.370 | Karkati 87 | AUS | *Indica* |
| 347 | 301337 | 0.420 | Creole | TRJ | *Japonica* |
| 348 | 301338 | 0.610 | China 1039 | IND | *Indica* |
| 349 | 301339 | 0.470 | Chang Ch'Sang Hsu Tao | IND | *Indica* |
| 350 | 301340 | 0.699 | Ligerito | TRJ | *Japonica* |
| 351 | 301393 | 0.716 | 68-2 | TEJ | *Japonica* |
| 352 | 301393 | 0.660 | Guatemala 1021 | TRJ | *Japonica* |
| 353 | 301341 | 0.240 | Arc 10376 | AUS | *Indica* |
| 354 | 301342 | 0.120 | *BALA | IND | *Indica* |
| 355 | 301343 | 1.350 | Asd 1 | TEJ | *Japonica* |
| 356 | 301344 | 0.280 | Jc 117 | IND | *Indica* |
| 357 | 301345 | 0.380 | 9524 | AUS | *Indica* |
| 358 | 301346 | 0.886 | 358 | ADMIX | *Japonica* |
| 359 | 301347 | 0.320 | Surjamkuhi | AUS | *Indica* |
| 360 | 301348 | 0.470 | Ptb 30 | AUS | *Indica* |
| 361 | 301350 | 0.959 | F.R. 13A | TEJ | *Japonica* |
| 362 | 301350 | 0.681 | Jamaica 3 | TRJ | *Japonica* |
| 363 | 301350 | 0.960 | Edomen Scented | TEJ | *Japonica* |
| 364 | 301351 | 0.676 | Rikuto Norin 21 | ADMIX | *Japonica* |
| 365 | 301352 | 0.982 | Shirogane | TEJ | *Japonica* |
| 366 | 301353 | 0.809 | Kiuki No. 46 | TEJ | *Japonica* |
| 367 | 301354 | 0.749 | Sanbyang-Daeme | ADMIX | *Japonica* |
| 368 | 301355 | 0.767 | Deokjeokjodo | TEJ | *Japonica* |
| 369 | 301356 | 0.140 | Sathi | AUS | *Indica* |
| 370 | 301357 | 0.120 | Coarse | AUS | *Indica* |
| 371 | 301358 | 0.490 | Santhi Sufaid | AUS | *Indica* |
| 372 | 301359 | 0.240 | Sufaid | AUS | *Indica* |
| 373 | 301360 | 0.440 | Lambayeque 1 | Group V | *Japonica* |
| 374 | 301396 | 0.859 | Benllok | TEJ | *Japonica* |
| 375 | 301396 | 0.799 | Upland | TRJ | *Japonica* |
| 376 | 301361 | 0.749 | Breviaristata | ADMIX | *Japonica* |
| 377 | 301362 | 0.751 | Pr 304 | TRJ | *Japonica* |
| 378 | 301363 | 0.170 | Kalubala Vee | AUS | *Indica* |
| 379 | 301364 | 0.594 | Wanica | TRJ | *Japonica* |
| 380 | 301365 | 0.891 | Tainan-Iku No. 512 | TEJ | *Japonica* |
| 381 | 301366 | 0.820 | 325 | TRJ | *Japonica* |
| 383 | 301398 | 0.820 | *COLL 2712 | TEJ | *Japonica* |
| 384 | 301367 | 0.940 | 318 | TRJ | *Japonica* |
| 385 | 301368 | 0.390 | Nira | IND | *Indica* |
| 386 | 301369 | 0.833 | Palmyra | ADMIX | *Japonica* |
| 387 | 301370 | 0.924 | M-202 | ADMIX | *Japonica* |
| 388 | 301371 | 1.011 | Nortai | ADMIX | *Japonica* |
| 389 | 301372 | 0.628 | Ci 11011 | ADMIX | *Japonica* |
| 390 | 301373 | 0.686 | CI 11026 | ADMIX | *Admixed* |
| 391 | 301374 | 0.786 | Della | TRJ | *Japonica* |
| 392 | 301375 | 0.714 | Edith | TRJ | *Japonica* |
| 393 | 301376 | 0.260 | *LA 110 | IND | *Indica* |
| 394 | 301377 | 0.688 | Lady Wright Seln | TRJ | *Japonica* |
| 395 | 301378 | 0.785 | Os 6 (Wc 10296) | TRJ | *Japonica* |
| 396 | 301379 | 0.490 | Cocodrie | TRJ | *Japonica* |
| 397 | 301380 | 0.920 | Cybonnet | TRJ | *Japonica* |
| 398 | 301399 | 0.317 | 9311 | IND | *Indica* |
| 399 | 301381 | 0.755 | Spring | TRJ | *Japonica* |
| 400 | 301400 | 0.339 | Yang Dao 6 | IND | *Indica* |
| 644 | 312013 | 0.412 | IR64 | IND | *Indica* |
