# Supplementary material for: Genetic Architecture of Aluminum Tolerance in Rice (Oryza sativa) Determined through Genome-Wide Association Analysis and QTL Mapping
Source: PLoS Genet. 2011 Aug 4;7(8):e1002221. doi: 10.1371/journal.pgen.1002221 (PMC3150440; doi:10.1371/journal.pgen.1002221)
Supplement: Table S2 — Evaluation criteria for selecting candidate SNPs based on P-values from EMMA within and across subpopulations and a priori knowledge of candidate genes. SNPs within a 200 kb window around 46 a priori candidate genes were considered a priori SNPs. Other SNPs were those that fell outside of the 200 kb window surrounding candidate genes, including those identified in the 23 QTL regions. (DOC) [file pgen.1002221.s007.doc]

**Table S2.**

| **P-value cut-off** | **Enrichment^*^** | **FDR_upper_%^$^** |
| --- | --- | --- |
| 1.E-03 | 1.17 | 0.86 |
| 1.E-04 | 2.39 | 0.42 |
| 1.E-05 | 2.81 | 0.36 |
| 1.E-06 | 2.48 | 0.40 |

*****Enrichment: (no. of *a priori* SNPs/no. of *other SNPs*) in the significant list/(no. of *a priori* SNPs/*other SNPs*) in non-significant list.

**$** FDR_upper_%: = 1−(x − y)/x = y/x. y is the fraction of non-*a priori* SNPs that are significant, and x is the fraction of *a priori* SNPs that are significant. Assume all non-*a priori* SNPs are false. FDR=false discovery rate.
